# Barriers and facilitators to detoxification from opioid substitution treatment: A mixed‐methods systematic review

**DOI:** 10.1111/add.70482

**Published:** 2026-06-17

**Authors:** Amy Bagshaw, Suleyman Shah, India Olchefske, Louise M. Paterson, Mike J. Crawford, Anne Lingford‐Hughes

**Affiliations:** ^1^ Division of Psychiatry, Department of Brain Sciences Imperial College London London UK

**Keywords:** addiction, barriers, detoxification, dose tapering, facilitators, methadone, opioid substitution treatment, systematic review

## Abstract

**Background and aims:**

Despite the well‐documented benefits of opioid substitution treatment (OST) in treating opioid dependence (OD), many people diagnosed with OD desire to live a drug‐free life. The transition to abstinence involves detoxification: a gradual dose reduction of OST to zero milligrams. Despite these aspirations, only a minority of patients undertake detoxification, and successful completion remains limited. This mixed‐methods systematic review aimed to identify the barriers and facilitators to detoxification from OST, to help provide a better understanding of what can be done to support patients and improve outcomes in OD.

**Methods:**

Four databases were searched until 22 January 2025: PubMed, Embase, APA PsycINFO and CINAHL. Qualitative, quantitative and mixed methods studies of individuals with a diagnosis of OD undergoing detoxification from OST, or staff responsible for providing the treatment, were included. Studies of only pharmacological interventions were excluded. An integrated approach to data synthesis was used, transforming quantitative data into textual descriptions to integrate them with qualitative data and form one set of themes. Joanna Briggs Institute checklists were used to assess the quality of included papers.

**Results:**

From 1999 studies identified, 41 papers were deemed eligible. Studies originated from the USA (22), UK (7), Sweden (6), Canada (1), Ireland (1), Norway (1), Switzerland (1), Australia (1) and China (1). The medications explored included methadone (28), buprenorphine (5), both forms (6) and unspecified OST (2). Studies were conducted in outpatient settings (29), inpatient settings (7), mixed settings (4) and a prison setting (1). Participants included patients (37), treatment providers (1) and mixed populations (3). Factors affecting detoxification were present at an individual and structural level, with overlap between influences at the initiation and completion phases. Eight themes emerged, comprising psychological and emotional factors (particularly around fear of detoxification), personal motivation, withdrawal symptoms, clinical and demographic factors, environmental factors, social factors, professional support and treatment models/interventions. 54% of included studies were rated high quality, and 37% of medium quality.

**Conclusion:**

Detoxification from opioid substitution treatment appears to be commonly hindered by fear, emotional resurgence, low confidence, environmental turbulence, negative social influences and insufficient professional/pharmacological support, while facilitators include psychological readiness, life stability, supportive relationships, psychological interventions, inpatient facilities and adjunctive medications.

## INTRODUCTION

Opioid dependence (OD) is a chronic relapsing disorder characterised by physiological dependence and a strong compulsion to use opioids, often leading individuals to prioritise drug use over other responsibilities or aspects of daily life [1]. The personal and public health burdens of OD are immense, with globally over 16 million people diagnosed with the disorder and over 120 000 opioid overdose deaths per year [2].

There is a wealth of evidence demonstrating that opioid substitution treatment (OST), combined with psychosocial support, improves wellbeing while reducing the financial and social strain of OD [3]. Methadone, a full opioid non‐selective agonist, and buprenorphine, a partial μ opioid agonist and κ antagonist, are both recommended by the World Health Organization (WHO) as first‐line treatments [4, 5]. These medications suppress cravings, reduce the risk of overdose and attenuate hazards associated with injecting [6]. However, despite the well‐documented benefits of OST, many patients aspire to live a life free of maintenance prescriptions, and in our clinical experience, free from all opioids [7].

Detoxification, or gradual dose reduction, plays a crucial role in the transition to abstinence. It is defined as a process of safe, supported tapering of a drug of dependence by a medical professional [8]. Given the benefits of OST, detoxification should not be imposed, and careful consideration and collaborative planning is a prerequisite. Detoxification support services are delivered in diverse settings, including in‐patient units (e.g. hospital, rehabilitation), criminal justice (e.g. prison) and, most frequently, outpatient clinics (e.g. community drug and alcohol services) [9].

Although many patients express a desire to be opioid‐free, relatively few actually attempt detoxification [10, 11, 12]. Among those who do begin the process, completion rates vary substantially, with most patients failing to reach abstinence [13, 14, 15, 16, 17, 18, 19]. The disconnect between patient aspirations, their attempts to taper and the variability in success, highlights a critical gap in our understanding. Factors that prevent patients from initiating detoxification and the challenges that lead to failed attempts remain unclear. This has significant clinical relevance as dose tapering carries substantial risks, including relapse to illicit opioid use and a sequelae of potentially life‐threatening consequences [20, 21].

To date, there have been no systematic reviews synthesising the barriers and facilitators to detoxification from OST. Understanding these factors would offer valuable insight into the steps necessary for preparation, and where attention should be focussed to improve outcomes. The aim of this mixed‐methods systematic review was, therefore, to identify factors that influence the initiation or completion of successful detoxification from OST, to provide a better understanding of what can be done to support patients and improve outcomes in OD. In this article, the term OST will be used to align with nomenclature commonly used in the United Kingdom (UK); however, it is acknowledged that ‘medication for opioid use disorder (MOUD)’, or ‘opioid agonist treatment’ (OAT), may be a preferred term in some communities.

## METHODS

### Design

This study was a narrative systematic review, following the Preferred Reporting Items for Systematic Reviews and Meta‐Analyses Protocols (PRISMA) guidelines (Appendix 4) [22]. To ensure all relevant studies were captured, a mixed‐methods approach was used. Findings of effectiveness of interventions (quantitative data) alongside patient and treatment provider experiences (qualitative data) were integrated to enhance the meaningfulness of evidence [23]. Several published reviews have explored optimum tapering schedules [16, 24] and the efficacy of pharmacological interventions to facilitate detoxification [25, 26, 27], so these studies were excluded.

The protocol for this study was prospectively registered at PROSPERO, registration number: CRD420250648944 (https://www.crd.york.ac.uk/PROSPERO/view/CRD420250648944).

### Search strategy

The following databases were searched: EMBASE, Medline, APA PsycInfo and CINAHL. The original search was conducted on 22 May 2024 and re‐run on 22 January 2025. Only papers published after 1965 were included as this was the first year methadone was prescribed as a substitution treatment for OD [28]. The final search strategies can be found in Appendix S1.

### Inclusion and exclusion criteria

The Patient Intervention Comparator Outcome (PICO) framework was used for this review. The population studied included: (1) individuals with an opioid addiction or dependence, defined by either engagement with a treatment service or formal diagnosis (e.g. Diagnostic and Statistical Manual of Mental Disorders/International Classification of Diseases), who are prescribed a variation of methadone or buprenorphine; and (2) treatment providers of detoxification for individuals with an opioid addiction (as defined). The intervention studied was detoxification—defined as the process of tapering OST until a 0 mg dose is reached—or dose reduction. There was no comparator. The outcomes of interest were factors like barriers or facilitators affecting the initiation or completion of detoxification.

Studies focusing solely on children under 16 years of age, non‐English publications, reviews, conference abstracts, dissertations, opinion pieces, letters, editorials, news items or case studies of one to two participants were excluded. Studies were also excluded if they only contained information about the efficacy of a pharmacological intervention (e.g. adjunctive medications, herbal remedies and on‐top illicit use) or dosing regimens (e.g. length of tapering), involved only participants with a prior dependence on prescription (e.g. oxycodone) or mild opioids (e.g. codeine) or evaluated rapid or ultra‐rapid detoxification procedures involving high‐dose naloxone with or without general anaesthesia or heavy sedation.

### Data extraction and synthesis

Following database searches, all search results were exported into Covidence and duplicate results removed [29]. Two reviewers (A.B., S.S.) independently screened each abstract to assess eligibility against pre‐defined inclusion and exclusion criteria, with discrepancies resolved through discussion with the third reviewer (A.L.H.). Included studies then underwent full‐text assessment and data extraction (A.B., S.S. and I.O.). The authors, country, participant group, sample size, setting, study design, OST type and taper length of each eligible study were extracted for tabulation (Appendix S2).

Studies with either a quantitative or a qualitative research design were included by using a convergent integrated approach, transforming data into the same format before synthesis [30]. Quantitative data were transformed into textual descriptions or narrative interpretations, then concurrently analysed and categorised with the qualitative data to form one set of themes. Although some barriers and facilitators were conversely appropriate, only one was reported.

### Quality assessment

Each study was assessed using the Joanna Briggs Institute (JBI) Quality Assessment checklists by a minimum of two reviewers (A.B., S.S. and I.O.) [31]. The checklist used was dependent on the study methodology, with scores standardised into a decimal value.

## RESULTS

### Study selection

**FIGURE 1:**
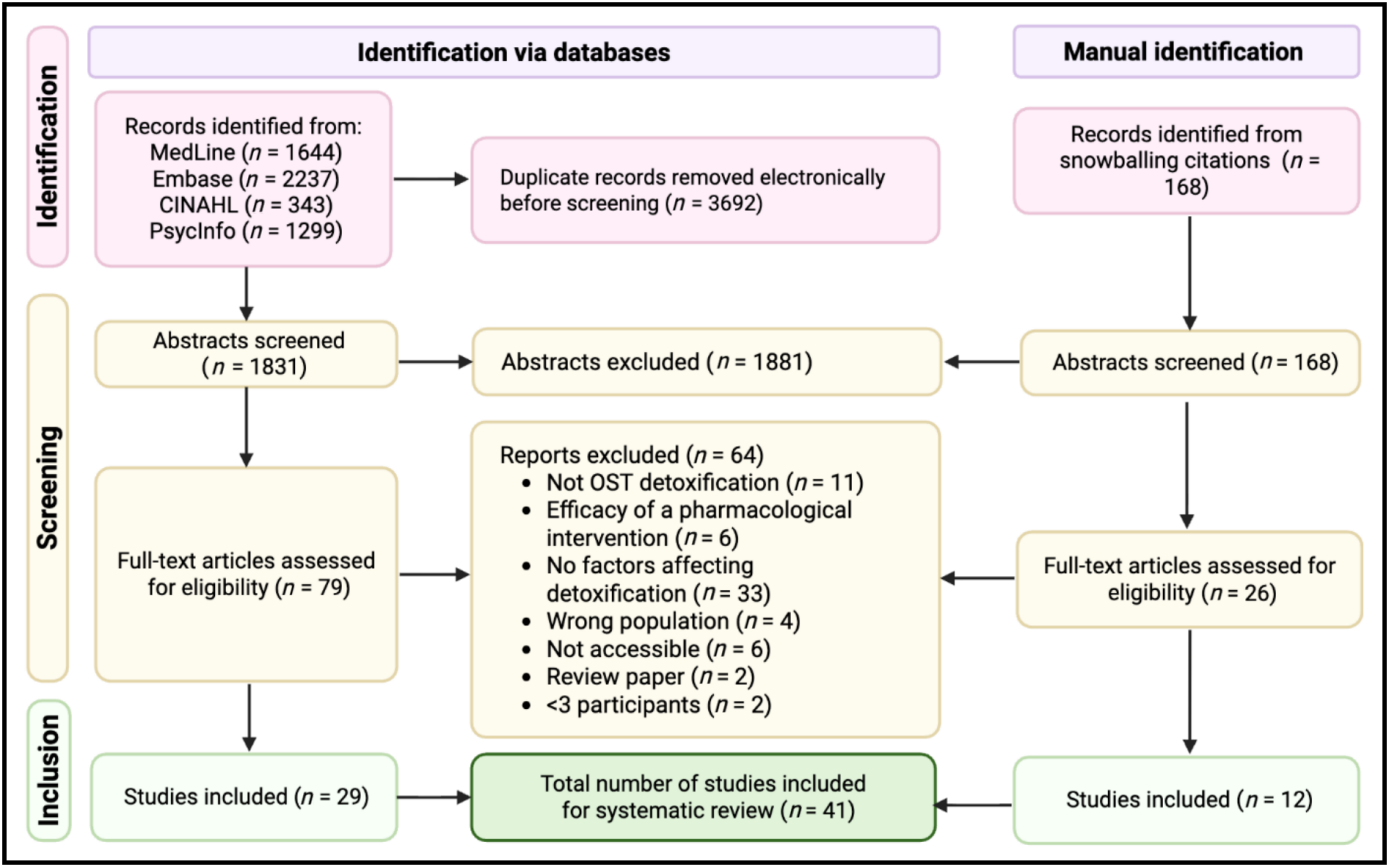
Preferred Reporting Items for Systematic Reviews and Meta‐Analyses (PRISMA) flowchart.

Following the removal of duplicates, the search strategy yielded 1999 unique records. After full text screening and a manual search of references, 41 papers were deemed eligible for data extraction (Figure 1).

### Characteristics of included studies

The 41 included studies were published between 1973 and 2024. Studies originated from the United States (US) (22), United Kingdom (7), Sweden (6), Canada (1), Ireland (1), Norway (1), Switzerland (1), Australia (1), and China (1). The medications explored included methadone (28), buprenorphine (5), both forms (6) and unspecified OST (2). Studies were conducted in outpatient settings (29), in‐patient settings (7), mixed settings (4) and a prison setting (1). Participants included patients (37), treatment providers (1) and mixed populations (3).

Regarding the primary illicit opioid of dependence in patient populations, studies stated heroin (19), inferred heroin (12) or did not specify (9). The methods of administration for primary illicit opioids (e.g. intravenous, inhalation) were rarely documented in the included studies. As such, these characteristics have not been reported in this review.

A summary of study characteristics included can be found in Appendix S2. A total of 22 studies were rated of high quality (score = >0.75); 15 were rated of medium quality (score = 0.50–0.75); and four were of low quality (score = <0.50) (Appendix S3). It should be noted that all studies rated low quality were published before 1997 when methodological norms and reporting standards were less rigorous. Although there was no correlation between quality ratings and study type, common methodological limitations were identified. Among qualitative studies, researchers frequently did not provide a statement situating themselves culturally or theoretically, nor did they describe their potential influence on the research process. Among quantitative studies, strategies to address incomplete follow‐ups and manage confounding variables were often not reported.

### Factors influencing detoxification

**FIGURE 2:**
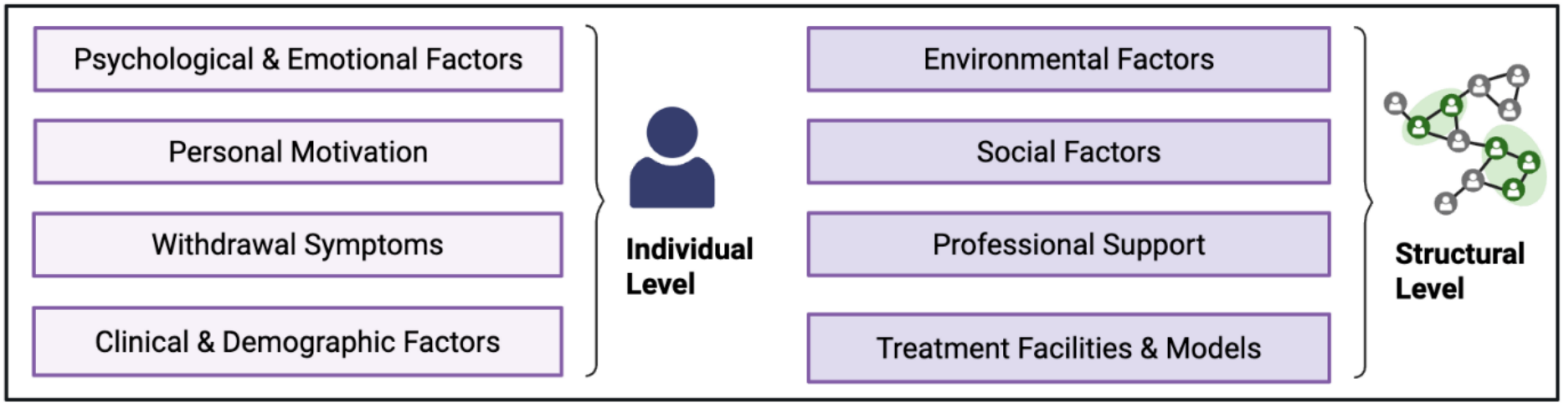
Summary of themes influencing detoxification from opioid substitution treatment (OST).

Analysis revealed that factors affecting detoxification occurred at individual and structural levels across eight themes, with overlap of factors influencing the initiation and completion of detoxification (Figure 2).

### Individual level

#### Theme 1: Psychological and emotional factors

##### Fear of the individual and emotional resurgence

In many studies, individuals reported fear as a prominent barrier to detoxification. This emotion manifested in several ways: fear of losing the stability that OST provided, fear of losing the support that the service provided, fear of repressed emotions resurfacing, fear of relapse to illicit opioids and fear of withdrawal symptoms—both physical and psychological [7, 11, 32, 33, 34, 35, 36, 37, 38, 39, 40]. These factors were primarily observed at the initiation phase, however, also seen during the dose taper, hindering successful completion:

‘I know how sick I will get. I would have quit long ago if I could. But I am scared to death about losing my maintenance treatment’ (patient) [34].

During the detoxification process, the experience of resurgence of emotions further disrupted tapering efforts—anxiety, stress, nervousness, mood swings and the resurfacing of feelings and traumatic memories that OST had played a role in numbing [10, 36, 38, 40, 41]. As doses lowered, patients struggled to cope with complex emotions leading many to feel destabilised [36, 38]:

‘I wasn't quite in reality anyway, and I could feel myself slowly coming down to reality, as I was dropping down even through the 5 mils…I could feel myself coming back with the touch, and with my feelings and everything coming back, you see the numbness out of your body going’ (patient) [38].

##### Psychological coping strategies

In contrast, psychological coping strategies were facilitators to both the initiation and completion of detoxification. These included the development of coping mechanisms, keeping one's mind busy and participation in hobbies that enhanced self‐esteem [36, 40]. Together, these tools helped patients to feel more prepared to handle the often‐turbulent process of detoxification:

‘Hope. The same way you handle a flu or a toothache. An addict falls into an instant gratification pattern. You realize that detoxing is not going to be painless. Reminding yourself how much you'll hate yourself for going back can be a big help. If you keep yourself busy, you'll stay out of trouble’ (patient) [36].

#### Theme 2: Personal motivation

##### Low self‐efficacy, desire and motivation

Several studies highlighted patients' lack of motivation to initiate detoxification. There was a desire to postpone the problem of dependency and avoid the need to face responsibilities OST had provided refuge from [11, 36]:

‘The fears and phobias that you originally tried to smother with heroin and later methadone are still there and you have to deal with them’ (patient) [36].

Many patients doubted their capability and readiness to achieve abstinence. These feelings were largely attributed to low personal efficacy, recovery capital, and quality of life [12, 36, 38, 42, 43]. This was particularly true in a prison setting, where incarceration and detox simultaneously was considered ‘overwhelming’ [41, 42]:

‘People don't [withdraw] voluntarily. None of these people have been detoxing on their own request’ (prison staff member) [42].

Low motivation was in part because of patient belief that OST was a necessity [10, 11]. Personal concern that they could not function without the medication was a prominent barrier to initiation. Content with the benefits methadone and buprenorphine were providing, particularly in relation to stability, several patients stated there was little incentive to undertake detoxification [36, 38, 42].

##### Personal motivation and desire for autonomy

Patients who initiated detoxification were often confident in their ability to achieve abstinence without relapsing to illicit opioids [10, 35]. The greater number of reasons a patient had to achieve abstinence, the higher the confidence in ‘readiness’, the ability to take personal responsibility and the willingness to participate in recovery programmes, all predicted successful outcomes [9, 10, 32, 33, 36, 38, 44].

There was a widespread aspiration to be liberated from OST, giving scope for personal growth, travel and economic opportunities [7, 10, 34, 36, 40, 45]. Many felt that they had outgrown the need for substitution medication, particularly if they were no longer using illicit substances, and longed to be free from physical dependence on any form of opioid [36, 39, 40, 46]:

‘Freedom! Free to be more than a LAR [medication assisted treatment] patient. To be free to travel. To visit my son in another part of Norway, even on short notice. Not to have to go to the pharmacy every day, even on Sundays. I'm completely fed up—I'm so fed up with taking medicine’ (patient) [40].

Patient dissatisfaction with OST was also a very clear motivator. This primarily centred on logistical concerns, such as attachment to a chemist and inconvenient visits to the service, and the desire to avoid side effects [7, 10, 34, 36, 45, 46]. Patients frequently began detoxification to end the hassle associated with maintaining OST and stigma surrounding it, particularly in relation to employment:

‘I'm afraid that I won't be able to get a job because of it [methadone]…If it wasn't stigmatized, I probably would stay on it’ (patient) [46].

In addition, a number of patients expressed that they were still experiencing cravings on buprenorphine, leading to opioid use and relapses while on medication. If OST was not providing the stability and benefits desired by the individual, the inconvenience of obtaining it did not appear justified [45]. Factors within this theme were equally observed in relation to both methadone and buprenorphine.

#### Theme 3: Withdrawal symptoms

##### Experience of withdrawal symptoms

Physiological withdrawal from OST was one of the most apparent barriers to the completion of detoxification, cited in 32% of papers [7, 9, 10, 14, 15, 18, 19, 36, 39, 40, 41, 47, 48]. The list of debilitating symptoms was extensive, including sleep disturbances, sickness, dysphoria, irritation, restlessness, pain, fatigue, malaise, narcotic hunger, lacrimation and gastrointestinal hyperactivity. These were all more severe at lower doses, with patients expressing a greater difficulty reducing at this point, leading to dissatisfaction with the dose of OST, intense cravings for opioids and no longer feeling ‘held’ [10, 18, 36, 39, 40, 48, 49, 50]:

‘Not only do you actually feel like you're going to die, or that you would like it to end, but you know there's only one thing on this planet that will make you feel better. And you can't have it’ (patient) [7].

In addition, patients highlighted a lack of comprehensive medical care to deal with the withdrawal. Without any adjunctive medication, the experience was considered ‘excruciating’ [7, 41].

##### Alleviation of withdrawal symptoms

Effective treatments to manage withdrawal were acknowledged by both patients and treatment providers as crucial in facilitating the transition from OST to abstinence [40]:

‘If I hadn't been given some special medicine to take away the tingling, I almost don't know if I would have lasted that long. And yes, I also got benzodiazepines’ (patient) [40].

This included both prescription medications (e.g. benzodiazepines) and alternative approaches, such as exercise, diet, massage, acupuncture and herbal teas [36, 40, 44, 51].

#### Theme 4: Clinical and demographic factors

Patients with a history of polydrug use before starting methadone treatment, higher methadone doses, co‐occurring mental health disorders, psychiatric instability and challenges with pain management were less likely to initiate or complete detoxification [11, 14, 38, 45, 52]. In contrast, successful outcomes were associated with, no history of injecting opioids, a longer period since last heroin use, lower methadone doses, fewer medical comorbidities and stronger adherence to treatment [13, 33, 39, 53, 54]. Lower stress and fewer sleep problems before initiating detox also facilitated successful transition to abstinence [47].

Some factors appeared to have differing influences depending on the stage of detoxification. A shorter duration of the current treatment episode or shorter history of opioid use was linked to increased likelihood of initiating detoxification [11, 35]. However, longer treatment duration on methadone or buprenorphine or a longer history of drug use, were reported to be associated with successful completion [45, 55].

Particular demographic characteristics were also related to outcomes: being male, welfare history, a middle‐class background, completing high school education and having children were all positively associated with the completion of a dose taper [13, 33, 44, 55]. One study also noted that previous contact with the criminal justice system did not prevent successful detoxification (32). However, findings on age were inconsistent. Some studies found younger individuals were more likely to achieve abstinence, whereas others found that older individuals were more likely to succeed [13, 33, 55, 56]. On balance, the number of studies for each of these factors in theme 4 is too small to draw firm conclusions.

### Structural level

#### Theme 5: Environmental factors

Key environmental barriers affecting both the initiation and completion of detoxification included poor housing, unemployment, ongoing life pressures and the lack of a drug‐free environment [15, 36, 38, 40, 41, 42, 52]. These challenges emerged in both community and prison contexts, often compounded by legal issues and broader environmental instability. In contrast, secure housing and steady employment tended to facilitate detoxification [18, 33, 42]. Successful completion was frequently associated with intentional changes to one's living situation—including relocation to a new residence or moving in with family—to foster a more supportive and drug‐free environment [36, 42, 55]:

‘The ones that I've seen do it ‘successfully’, the ones I haven't seen come back, have received outside support in the form of successful work situations and successful family situations like their spouse, parents, friends and they got out of their environments’ (staff member) [36].

Temporary practical arrangements, like taking time off work or securing childcare during detoxification, also contributed to positive outcomes [44].

#### Theme 6: Social factors

Patients who were more involved in, and satisfied with, the drug culture or content with their current drug use situation, were less likely to pursue detoxification [11]. After years of being involved with drug culture, some individuals expressed that their identity and the culture were intertwined [38]:

‘I have taken drugs all my life; it has just escalated from C to B to A as I do, and you know, I probably will always use heroin’ (patient) [38].

Lack of a supportive network, pressure from family or friends to remain on methadone for fear of relapse and continued association with drug‐using peers further hindered motivation to begin and complete the process [9, 11, 15, 32, 38, 40]. Individuals were hesitant to consider detoxification as a treatment avenue having known peers who had unsuccessfully attempted to quit [11].

Conversely, social reintegration, particularly with non‐drug‐using individuals, and exposure to successful role models of detoxification were associated with initiation and completion of detoxification [10, 36]. A strong support system, new social associations, involvement in self‐help groups and support or pressure from family and friends were all beneficial [36, 40, 46]:

‘They [peers who had successfully detoxed] are constant source of inspiration and confidence for me. You hear so many stories of how impossible it is to detox. It's nice to see the real thing in front of you. They were successful because they desired to be free of methadone, and it was time to get complete control of their lives’ (patient) [36].

The value of peer support was also apparent, where groups of individuals could undertake the detoxification process together and positively encourage each other [38, 42]:

‘Upstairs…people come in, ‘oh, why don't you go and get this…they'll sort you out [with drugs]’. Down here, they were coming in and talking to me [when] I was lying on the bed. ‘You know, you'll be alright” (prison patient) [42].

#### Theme 7: Professional support

Several studies emphasised the critical role of professional support for detoxification at both initiation and completion stages. Barriers primarily centred around the lack of psychosocial and psychological provisions in both community and prison settings [34, 36, 41, 42]. This was of particular importance during the final stage of the dose tapering where fear, withdrawal and mental instability were at their greatest [34, 40, 41]:

‘I need help for my background anxiety problems…But the only thing they offer is antidepressants. I have been offered the whole list of them…They focus too much on medication, and that is the wrong focus. There is so much focus on medication, rather than on the underlying problems or that you need to get on with your life…I feel as if they are in total control of my life’ (patient) [34].

Patients also reported that staff frequently did not advocate for detoxification. When expressing a desire for abstinence, they were often met with dissuasion from counsellors who did not think it was possible to leave OST and remain consistently drug‐free [34, 40, 42]:

‘They always told me ‘You're going to have this for the rest of your life’. And when I decided to quit, they said I was never going to make it…But I felt ‘damn, I'm going to show them!’ I knew they had no right to stop me from quitting’ (patient) [34].

One study reported that patients were more likely to complete detoxification if treatment staff were unaware of their withdrawal attempt [44]. Those responsible for professionally overseeing the detox were not always seen as suitably trained or competent from the perspective of patients, often displaying a lack of understanding and empathy for those undergoing a dose taper [41]:

‘The nurses knew nothing about when I was being tapered down, they didn't come and check. Nobody came to check nothing. They just sat there and said ‘suck it up, it's not that bad’. The medical staff is horrible…their opinion is, you can't die from opiate withdrawal so suffer.’ (prison patient) [41].

Conversely, patients stated that continuous discussions with treatment providers, particularly those who acknowledged the challenges associated with detoxification,  facilitated initiation [10, 34, 35, 42]. Patients expressed that they did not want to feel pressure to detox, but instead wanted to be supported regardless of their decision [34]. If an individual decided to proceed with detoxification, professional psychological interventions, in both individual and group settings, were beneficial in sustaining tapering effort [36, 38, 42]:

‘Periodic counselling would be beneficial for me. She (counsellor) makes me feel like she understands me. She really is concerned. For me, that's a definite plus’ (patient) [36].

Moreover, establishing a strong, open and supportive relationship with treatment staff appeared to be crucial, with outcomes particularly favourable when detoxification was led by psychiatrists or psychologists and included input from former service users [9, 33, 40, 57].

#### Theme 8: Treatment facilities and models

Treatment models and approaches linked to successful detoxification included accessible one‐to‐one support structures, small cohorts in treatment, incorporation of behavioural interventions such as voucher incentive programs, engagement in therapeutic activities and a community reinforcement approach [9, 42, 58].

Granting patients greater control over their detoxification process also emerged as a key facilitator to completion. Allowing flexibility to pause or temporarily increase the tapering dose was associated with improved outcomes, with gradual, self‐paced reduction generally proving more effective [10, 13, 21, 34, 36, 40, 59]. However, findings were not entirely consistent: one study reported better outcomes with abrupt withdrawal, whereas another suggested that a fixed, non‐negotiable schedule could be more effective [44, 54].

In‐patient facilities were also frequently noted as beneficial, either as a setting to complete the dose reduction or as part of a structured post‐detoxification plan [9, 15, 53, 56, 57]. These facilities provided patients environmental respite to focus on OST tapering without external distractions:

‘I felt more protected here than when doing a home detox. There were no outside influences’ (patient) [9].

Subjects who expressed a clear preference for detoxification setting, either in‐patient or outpatient, were also more likely to reach abstinence [15]. Nonetheless, limited service capacity and long waiting times continue to pose significant barriers to accessing such support [60].

## DISCUSSION

The aim of this systematic review was to provide a better understanding of what can be done to support individuals with an aspiration to be ‘free from OST’, yet struggle to achieve this goal. Across studies both initiation and completion phases of detoxification were frequently hindered by fear, resurgence of emotions, lack of confidence, environmental turbulence, negative social influence and inadequate support—both professional and pharmacological. In contrast, facilitators to the process included psychological readiness, life stability, supportive relationships —with friends, family and those working in addiction services—, psychological interventions, in‐patient facilities and adjunctive medications. These findings were largely consistent across time, with earlier and more recent papers identifying similar key factors.

Over the last few decades, there has been a shift away from detoxification and abstinence‐only models to those of longer‐term engagement, harm reduction and provision of OST. Therefore, the availability and delivery of detoxification, including appropriate support, has consequently changed. Findings from this review suggest that barriers to detoxification remain insufficiently addressed. Emergent themes underlie the importance of a wraparound, tailored approach to treatment, which incorporates psychological and pharmacological therapies and addresses broader social and environmental issues to provide patients with the tools to succeed in their chosen avenue of treatment for OD. However, it is important to note that tapering off OST is not a treatment goal for many patients, and the desire for ongoing use of medication that benefits stability and mitigates the risk of mortality should be respected.

### Lack of patient‐centred care

In recent years, patient‐centred care has been at the forefront of the treatment narrative for OD. For instance, in the United Kingdom, Dame Carol Black's ‘Independent Review of Drugs’ advocated for individual choice and personalisation of care [61, 62]. This approach relies on a strong therapeutic relationship, characterised by collaboration, trust and shared decision‐making [63]. Indeed, good evidence has shown that the quality of therapeutic alliance improves treatment engagement and outcomes across a range of mental health and substance use disorders [63, 64, 65, 66]. However, the findings of this review suggest that this approach is not always being translated into practice. Multiple studies highlighted unsupportive staff, inadequate support and rigid detoxification schedules as barriers to the withdrawal process [34, 36, 40, 41, 42]. These findings suggest that similar barriers to detoxification from OST exist across other countries as well.

### Fear and psychological interventions for preparation

It is important to note that although fear was a prominent barrier to detoxification, this is not unique to opioids. Several studies exploring alcohol and benzodiazepine withdrawal have revealed patient concern around physical dangers (e.g. seizures, delirium), and although cocaine withdrawal is not as physically severe, individuals face similar challenges navigating emotional symptoms [67, 68, 69]. However, completion rates of withdrawal from other substances are consistently greater than those observed in opioid dependent populations [70]. Fear around OST detoxification appears to be much more profound and multi‐faceted. This may be partially explained by the dysregulation of hedonic processes that drive motivation and reward, the elevated risk of fatal overdoses following relapse and the longer, more entrenched treatment history of the patient population [2, 70, 71]. Fear may be further exacerbated by the real prospect of losing the psychosocial support and structure that is provided by the regular service engagement that surrounds the provision of OST. The findings of this review suggest that fear around the initiation of detoxification is not being adequately addressed to prepare patients to undertake the process.

Psychological interventions have served to alleviate certain fears surrounding detoxification from drugs of dependence by guiding individuals through what to expect during the process [72]. This is a crucial part of treatment preparation that equips patients to navigate the withdrawal experience and challenges that come with it. However, although training for provision of basic interventions (e.g. motivational interviewing) appears to be high within drug and alcohol workers, it is not a standardised or regulated practice [73, 74]. It remains unclear what exact training is provided and whether or not it is adequate. In the United Kingdom, the number of regulated psychological professionals within addiction services is low, accounting for just 2% of the workforce [75]. There may not be enough highly trained staff to prepare and guide individuals through the emotionally turbulent process of detoxification or to adequately support them in early abstinence.

### Withdrawal symptoms and therapeutics

The physical experience of withdrawal symptoms presents a significant challenge to detoxification from OST. At a symptomatic level, benzodiazepines are prescribed for anxiety and restlessness, antidepressants or antihistamines for insomnia and non‐steroidal anti‐inflammatories for muscle aches [76]. However, these adjuncts provide limited relief and many have been associated with tolerance or abuse liability [77]. Lofexidine, an α‐2 adrenergic agonist, remains the only drug licensed to support multiple symptomatic facets of opioid withdrawal with notable success [78, 79, 80]. However, for reasons that remain unclear, lofexidine is no longer available in the United Kingdom yet remains licensed in the United States [77, 81]. There are no alternative licensed non‐opioid medications that target the underlying neurobiology of withdrawal, exposing a critically unmet need in the treatment of OD. This area has seen little innovation in recent decades, and the identification of novel therapeutics is essential.

### Environmental respite and social support

Research has long established that having somewhere safe and stable to live equips individuals to take on additional work around self‐improvement [61]. Indeed, the importance of environmental respite is clearly reflected in the findings of this review, including the benefits provided by in‐patient or residential facilities, either for the dose taper or as part of a post‐detoxification plan. This is a vital treatment avenue for clients with complex needs, or for those who do not have the resources or surroundings conducive to success [8]. However, the number of available places has declined in recent decades because of budget cuts, and demand is outstripping supply [61, 82]. More funding needs to be made available to improve capacity of specialised in‐patient services for those who would benefit from this treatment approach [61].

In line with our findings, there is also good evidence to suggest that patients with strong social support networks show improved treatment outcomes [83]. Such connections help individuals to foster a sense of accountability, to deal with the emotional consequences of addiction and to tackle issues with isolation [84, 85, 86]. It is, therefore, crucial that individuals have the opportunity to establish new, recovery‐orientated connections that can support the transition to abstinence should this not already be in place. There are several ways through which services can facilitate the formation of such relationships, including group work, social prescribing or peer‐support [87, 88, 89, 90]. These interventions provide an opportunity for interactions with non‐drug using populations and may help to encourage behaviours conducive to recovery. Moreover, services could help to signpost patients to other sources of support following detoxification from OST, such as abstinence‐based lived experience recovery organisations (LERO), to facilitate transition to a non‐service phase of treatment. However, there is no national or global data documenting the uptake of these services and whether or not they are adequate [91].

### Strengths and limitations

One of the key strengths of this systematic review is the combination of qualitative and quantitative methodologies included. Given the paucity of data available, this is the first review exploring barriers and facilitators to detoxification and encompasses all available literature on the topic. However, this mixed methodology also poses challenges in terms of merging codes in the analysis to ensure they naturally fit under a theme. The factors affecting initiation and completion of detoxification may also vary dependent on the form of OST prescribed given their differing pharmacological profiles. The findings of this review were largely biased toward methadone detoxification given the greater number papers stemming from its longer historical use as a maintenance treatment. As such, it was not possible to disentangle what barriers were unique to each formulation. Moreover, there was an evident lack of studies exploring factors influencing detoxification from the perspective of treatment providers, with fewer than 10% of eligible studies recruiting this population. Such research would provide insight into the structural and service‐level barriers to OST tapering, which have not yet been adequately addressed in the literature. Finally, although not within the remit of this review's inclusion criteria, an evident barrier to detoxification was the ongoing use of illicit opioids [37, 50, 58, 92]. Several papers further explored interventions through which on‐top use could be attenuated, however, no reviews have yet been conducted to synthesis the evidence [58, 93, 94, 95, 96].

### Implications

Whilst acknowledging that the cessation of OST needs careful consideration by both the individual with OD and treatment service team, our experience currently in the United Kingdom, is that detoxification is not routinely offered. For instance, the number leaving treatment ‘free of OD’ in England has been falling over the last decade or so from approximately 37% to approximately 23% [70, 97]. This review suggests a range of measures that should be considered urgently by services to respond to an individual's request to be opioid‐free and improve outcomes. Many of these are routine in alcohol detoxification pathways, but less often present for opioid detoxification, likely because of the focus on OST. These factors include the individual having a degree of control over their taper, staff adequately trained in managing opioid withdrawal, support and advice from those who have successfully completed detoxification (e.g. peer support workers), adjunctive psychological support to overcome issues with fear, pharmacological medications to alleviate the emergence of withdrawal symptoms and greater availability of in‐patient or residential settings to provide a conducive, stable environment for those without suitable surroundings.

## CONCLUSION

In conclusion, detoxification from OST remains a significant challenging with limited success, but clear pathways toward better treatment provision to support those undertaking this process can be facilitated. The findings of this review indicate that several strategies should be urgently considered, by both patients and services, to optimise treatment outcomes in those who aspire to live an opioid‐free life.

## AUTHOR CONTRIBUTIONS

**Bagshaw A:** Writing—original draft; methodology; project administration; formal analysis; conceptualization; investigation; data curation; validation; visualization. **Shah S:** Writing—review and editing; methodology; investigation; data curation; validation. **Olchefske I:** Writing—review and editing; investigation; data curation; validation. **Paterson LM:** Supervision; writing—review and editing; conceptualization. **Crawford MJ:** Writing—review and editing; supervision. **Lingford‐Hughes A:** Supervision; writing—review and editing; methodology; conceptualization; formal analysis.

## DECLARATION OF INTERESTS

A.L.H. has received honoraria paid into her institutional funds for speaking and chairing engagements from Lundbeck, Lundbeck Institute UK, Janssen‐Cilag, Pfizer, Servier; received research grants or support from Lundbeck, GSK and Indivior; unrestricted funds support from Alcarelle for a PhD; consulted by Silence, NET Device Corps, Sanofi‐Aventis, Astra Zeneca, Indivior, Neurocentrix, Eli Lilly and also consulted by but received no monies from Britannia Pharmaceuticals, GLG, Opiant, Lightlake, Dobrin, Nodthera and Libero. She is Chair of Addiction Healthcare Goals, Office for Life Sciences. L.M.P. has done consultancy work for Indivior (paid) and GABA Labs (formerly Alcarelle) (unpaid). All other authors have no declaration of interests to report.

## STUDY REGISTRATION

Prospero registration number CRD420250648944.

## Supporting information


**Appendix S1.** Supplementary Information.

**Appendix S2.** Supplementary Information.

**Appendix S3.** Supplementary Information.

**Appendix S4.** Supplementary Information.

**Appendix S5.** Supplementary Information.

## Data Availability

The data that supports the findings of this study are available in the supplementary material of this article (Appendix S5).
