# Supplementary material for: Barriers and facilitators to detoxification from opioid substitution treatment: A mixed‐methods systematic review
Source: Addiction. 2026 Jun 17;121(10):2607–19. doi: 10.1111/add.70482 (PMC13578861; doi:10.1111/add.70482)
Supplement: Supplementary file 1 [file ADD-121-2607-s001.docx]

**APPENDIX 2: DETAILS OF STUDIES INCLUDED**

| **Author/Year** | **Country** | **Participants** | **Sample Size** | **Setting** | **Design** | **OST** | **Taper Length** |
| --- | --- | --- | --- | --- | --- | --- | --- |
| Mintz et al., 1975 | USA | OUD patients | 24 participants (12 experimental, 12 control) | Outpatient | Randomised controlled trial | Methadone | 14 weeks |
| Wyse et al., 2023 | USA | OUD patients | 14 participants | Outpatient | Qualitative interviews | Buprenorphine | Not applicable |
| Hakansson & Hallen, 2014 | Sweden | OUD patients | 122 participants | Inpatient | Case series | Buprenorphine | x̄ = 13.3 days |
| Shaw, 2010 | UK | OUD patients | 17 participants | Inpatient | Cohort, qualitative interviews | Methadone, Subutex | x̄ = 18.1 days |
| Fulwiler et al., 1979 | USA | OUD patients | 30 participants | Outpatient | Randomised controlled trial | Methadone | 16 days |
| Calsyn et al., 2006 | USA | OUD patients | 30 participants | Outpatient | Case series | Methadone | x̄ = 10.9 months |
| Page et al., 2016 | UK | OUD patients, treatment providers | 196 participants (102 patients, 94 treatment providers) | Prison | Qualitative interviews | Not specified | Not applicable |
| Gossop et al., 1987 | UK | OUD patients | 116 participants | Inpatient | Case control | Methadone | 21 days |
| Berger & Schwegler., 1973 | USA | OUD patients | 19 participants | Outpatient | Case series | Methadone | x̄ = 47 days |
| Broers et al., 2000 | Switzerland | OUD patients | 73 participants | Inpatient | Case series | Methadone | x̄ = 15.3 days |
| Levin et al., 1997 | USA | OUD patients | 19 participants | Inpatient | Cohort | Methadone | 4 days |
| Nehlin et al., 2022 | Sweden | OUD patients | 15 participants | Outpatient | Qualitative interviews | Methadone, Buprenorphine | Not applicable |
| Mullen et al., 2010 | Ireland | OUD patients | 327 participants | Inpatient, outpatient | Case series | Methadone | 12 weeks |
| Winstock et al., 2011 | Australia | OUD patients | 145 participants | Outpatient | Analytical cross-sectional | Methadone, Buprenorphine | Not applicable |
| Bickel et al., 1997 | USA | OUD patients | 39 participants (19 experimental, 20 control) | Outpatient | Randomised controlled trial | Buprenorphine | 26 weeks |
| Gold et al., 1988 | USA | OUD patients, treatment providers | 90 participants (60 patients, 30 treatment providers) | Outpatient | Qualitative interviews | Methadone | Not applicable |
| Milby et al., 1994 | USA | OUD patients | 271 participants | Outpatient | Cohort, qualitative interviews | Methadone | Not applicable |
| Manelli et al., 2022 | USA | OUD patients | 101 participants | Outpatient | Analytical cross-sectional | Buprenorphine | Not applicable |
| Kanof et al., 1993 | USA | OUD patients | 24 participants | Outpatient | Case series | Methadone | Not specified |
| Dawe et al., 1991 | UK | OUD patients | 39 participants | Outpatient | Randomised controlled trial | Methadone | 6 weeks |
| Wermuth et al., 1987 | USA | OUD patients | 113 participants (57 experimental, 56 control) | Outpatient | Case-control | Methadone | Not specified |
| Hiltunen & Eklund, 2002 | Sweden | OUD patients | 50 participants (25 experimental, 25 control) | Outpatient | Analytical cross-sectional | Methadone | Not applicable |
| Stewart et al., 2000 | UK | Treatment providers | 54 drug treatment programmes | Inpatient, outpatient | Analytical cross-sectional | Methadone | Not applicable |
| Cushman, 1974 | USA | OUD patients | 118 participants | Outpatient | Case series | Methadone | Not specified |
| Kahn et al., 1976 | USA | OUD patients | 63 participants | Outpatient | Case series | Methadone | 21 days |
| Nesvag et al., 2024 | Norway | OUD patients | 24 participants | Outpatient | Qualitative interviews | Not specified | Not applicable |
| Aronowitz & Laurent, 2016 | USA | OUD patients | 10 participants | Outpatient | Qualitative interviews | Methadone, Suboxone | Not applicable |
| Lu et al., 2024 | China | OUD patients | 116 participants (60 experimental, 56 control) | Outpatient | Randomised controlled trial | Methadone | 8 weeks |
| Hiltunen et al., 2011 | Sweden | OUD patients | 38 participants | Outpatient | Cohort | Methadone | Not applicable |
| Notley et al., 2015 | UK | OUD patients, treatment providers | 37 participants (27 patients, 10 treatment providers) | Outpatient | Qualitative interviews | Methadone, Buprenorphine | Not applicable |
| Eklund et al., 1995 | Sweden | OUD patients | 50 participants | Outpatient | Analytical cross-sectional | Methadone | Not applicable |
| Senay et al., 1977 | USA | OUD patients | 127 participants (63 experimental, 64 control) | Outpatient | Cohort | Methadone | 30 weeks |
| Raynes et al., 1973 | USA | OUD patients | 149 participants | Inpatient | Cohort | Methadone | Self-determined x̄ = 22.3 days, physician control x̄ = 18.5 days |
| Gossop et al., 1986 | UK | OUD patients | 60 participants | Inpatient, outpatient | Cohort | Methadone | Outpatient = 8 weeks, inpatient = 3 weeks |
| Cushman & Dole, 1973 | USA | OUD patients | 48 participants | Inpatient | Case series | Methadone | Not specified |
| Stimmel et al., 1978 | USA | OUD patients | 429 participants | Outpatient | Case series | Methadone | Not specified |
| Eklund et al., 1997 | Sweden | OUD patients | 20 participants (10 experimental, 10 control) | Outpatient | Case-control | Methadone | Not specified |
| Nosyk et al., 2012 | Canada | OUD patients | 14,602 participants | Outpatient | Cohort | Methadone | Not specified |
| Chappel et al., 1973 | USA | OUD patients | 359 participants (244 experimental, 115 control) | Inpatient, outpatient | Cohort | Methadone | Not specified |
| Stein et al., 2019 | USA | OUD patients | 138 participants | Outpatient | Qualitative interviews | Buprenorphine | Not applicable |
| Randall-Kosich et al., 2020 | USA | OUD patients | 31 participants | Outpatient | Qualitative interviews | Methadone, Buprenorphine | Not applicable |
