# Supplementary material for: Barriers and facilitators to detoxification from opioid substitution treatment: A mixed‐methods systematic review
Source: Addiction. 2026 Jun 17;121(10):2607–19. doi: 10.1111/add.70482 (PMC13578861; doi:10.1111/add.70482)
Supplement: Supplementary file 2 [file ADD-121-2607-s002.docx]

**APPENDIX 3**

**Table 2:** **JBI Checklist Output**

| **Author/Year** | **JBI Checklist Type** | **Standardised Score** | **Quality Rating** |
| --- | --- | --- | --- |
| Mintz et al., 1975 | Randomised controlled trial | 0.92 | High |
| Wyse et al., 2023 | Qualitative | 0.70 | Medium |
| Hakansson & Hallen, 2014 | Case series | 0.90 | High |
| Shaw, 2010 | Cohort, qualitative | 0.71 | Medium |
| Fulwiler et al., 1979 | Randomised controlled trial | 0.82 | High |
| Calsyn et al., 2006 | Case series | 0.90 | High |
| Page et al., 2016 | Qualitative | 0.60 | Medium |
| Gossop et al., 1987 | Case control | 0.90 | High |
| Berger & Schwegler, 1973 | Case series | 0.80 | High |
| Broers et al., 2000 | Case series | 0.90 | High |
| Levin et al., 1997 | Cohort | 0.64 | Medium |
| Nehlin et al., 2022 | Qualitative | 0.70 | Medium |
| Mullen et al., 2010 | Case series | 0.80 | High |
| Winstock et al., 2011 | Analytical cross-sectional | 0.88 | High |
| Bickel et al., 1997 | Randomised controlled trial | 0.64 | Medium |
| Gold et al., 1988 | Qualitative | 0.60 | Medium |
| Milby et al., 1994 | Cohort | 0.80 | High |
| Manelli et al., 2022 | Analytical cross-sectional | 0.75 | High |
| Kanof et al., 1993 | Case series | 0.80 | High |
| Dawe et al., 1991 | Randomised controlled trial | 0.62 | Medium |
| Wermuth et al., 1987 | Case control | 0.44 | Low |
| Hiltunen & Eklund, 2002 | Analytical cross-sectional | 0.88 | High |
| Stewart et al., 2000 | Analytical cross-sectional | 1.00 | High |
| Cushman, 1974 | Case series | 0.80 | High |
| Kahn et al., 1976 | Case series | 0.40 | Low |
| Nesvag et al., 2024 | Qualitative | 0.90 | High |
| Aronowitz & Laurent, 2016 | Qualitative | 0.90 | High |
| Lu et al., 2024 | Randomised controlled trial | 0.92 | High |
| Hiltunen et al., 2011 | Cohort | 0.75 | High |
| Notley et al., 2015 | Qualitative | 0.70 | Medium |
| Eklund et al., 1995 | Analytical cross-sectional | 0.38 | Low |
| Senay et al., 1977 | Cohort | 0.80 | High |
| Raynes et al., 1973 | Cohort | 0.46 | Low |
| Gossop et al., 1986 | Cohort | 0.64 | Medium |
| Cushman & Dole, 1973 | Case series | 0.70 | Medium |
| Stimmel et al., 1978 | Case series | 0.70 | Medium |
| Eklund et al., 1997 | Case control | 0.70 | Medium |
| Nosyk et al., 2012 | Cohort | 0.80 | High |
| Chappel et al., 1973 | Cohort | 0.60 | Medium |
| Stein et al., 2019 | Qualitative | 0.60 | Medium |
| Randall-Kosich et al., 2020 | Qualitative | 0.80 | High |
