# Supplementary material for: Barriers and facilitators to detoxification from opioid substitution treatment: A mixed‐methods systematic review
Source: Addiction. 2026 Jun 17;121(10):2607–19. doi: 10.1111/add.70482 (PMC13578861; doi:10.1111/add.70482)
Supplement: Supplementary file 4 [file ADD-121-2607-s004.docx]

**Title:** Barriers and Facilitators to Detoxification from Opioid Substitution Treatment: A Mixed-Methods Systematic Review

**Authors:** Bagshaw A^a^, Shah S^a^, Olchefske I^a^, Paterson LM^a^, Crawford M^a^, Lingford-Hughes A^a^

^a^ Division of Psychiatry, Department of Brain Sciences, Imperial College London

**1A. Barriers to Initiation**

- Fear of return of emotions that Buprenorphine had blunted (e.g., one participant feared they may return to depression “being a recluse, not wanting to talk, being cold.” (Wyse et al., 2023). Fear of return of negative emotions.
- In Type A (comprehensive clinical services, 46% of prison population) and Type B (selective clinical, 5% of prison population) Drug Recovery Wings, many prison residents do not want to detox. Psychosocial workers struggled to find abstinence- minded opioid substitution therapy recipients. ‘People don’t [withdraw] voluntarily. None of these people have been detoxing on their own request’ (Staff, Type A). (Page et al., 2016). Low desire for detoxification.
- In Type A and Type B DRW, patients had low recovery capital that is not supported (nearly all had experienced extensive homelessness, and most entered Type A DRWs whilst vulnerable and withdrawing). Clinical staff described prisoners arriving malnourished, with dire mental health, dental problems, and ‘a lot of wounds that have just been left’ (Staff, Type B). Opportunities to build recovery capital were also sparse, consisting of access to the gym and occasional cookery classes. In this context, senior staff had come to see abstinence as highly ambitious: ‘I don’t think [recovery] means abstinence because I’m not sure how realistic [that is]’ (Staff, Type B). Low recovery capital that is not supported.
- Treatment was sparse in Type A DRW. Residents could access one psychosocial one-to-one each month. Neither DRW delivered any group programmes (or interventions focused on building recovery capital), and several prisoners identified medication as the only meaningful support. ‘I: What have the most helpful things been? R: The methadone, really. I: Anything else... you’ve found helpful? R: Not really (Prisoner, Prison 1). Lack of psychosocial support.
- DRW staff routinely advocated for sustained prescribing over detoxification. Staff not advocating for detox of clients.
- Some prisoners were fraught at the prospect of reduction. One stated, "Without [Subutex] I’d hurt myself and cut my wrists, because I can’t cope without it" (Prisoner, Type A).’ Fear of not having opioid substitution therapy medication.
- The increased availability of drugs thus made abstinence more challenging for vulnerable residents, whilst the black-market value of medication may have discouraged some others from detoxification. Staff certainly believed many residents actively sought imprisonment whilst ‘packed up [with drugs] to sell’ (Staff, Type A), and one prisoner reflected: ‘They’re running around, swapping their scripts. The majority of them, all they’re interested in is getting out of their nut’ (Prisoner, Type A). Availability of illicit drugs/lack of drug-free environment.
- All used the word “fear” in describing their reactions to being in a drug-free environment (Berger & Schwegler, 1973). Fear of not having opioid substitution therapy medication.
- Some expressed that the staff were reluctant or even negative about the process of tapering off treatment: ‘I need help for my background anxiety problems. (...) But the only thing they offer is antidepressants. I have been offered the whole list of them. (...) They focus too much on medication, and that is the wrong focus. There is so much focus on medication, rather than on the underlying problems or that you need to get on with your life. (...). I feel as if they are in total control of my life.’ (Nehlin et al., 2022). Lack of psychosocial support.
- A few participants had a wish to come off the treatment which was not supported by the program staff. “They always told me, ‘You’re going to have this for the rest of your life.’ And when I decided to quit, they said I was never going to make it. (. . .) But I felt ‘damn, I’m going to show them!’ I knew they had no right to stop me from quitting”. Detox not supported by staff despite patient desire.
- Some feared that they would be left alone if they managed to taper off and called for prolonged care after detoxification – “Once you have tapered out, that’s when the hard part begins. But at that time, they are not responsible for you anymore. (. . .) There must simply be a program for those who want to leave. Something with at least a streak of science in it”. (#12) Fear of being left alone after detox.
- Some participants expressed fear of not having medication. “I know how sick I will get. I would have quit long ago if I could. But I am scared to death about losing my maintenance treatment. (#12).” Fear of not having opioid substitution therapy.
- Primary concerns with detoxification were fear of withdrawal discomfort (68%, n = 99), fear of increased pain (50%, n = 72), return to heroin use (48%, n = 69), “life becoming a mess” (34%, n = 50), return to crime (30%, n = 44), loss of contact with clinic (17%, n = 24). Only 5 subjects (3%) identified concerns about overdose (Winstock et al., 2013).
- Fear of returning to illicit drug use. 'it’s the steadiest supply. No withdrawal symptoms, and I’m comfortable with my dose. I find it is stabilizing in terms of lifestyle...Methadone maintenance makes life more stable, less tiring. I don’t want to suffer withdrawal symptoms and if I get off of methadone maintenance, I’ll go back to opiates. (Gold et al., 1988).
- Fear of psychological difficulties - fear/ anticipation of feeling sick, irritable, in pain, anxiety, stress, nervousness, mood swings, resurfacing of feelings, and having one’s mind constantly occupied with tapering. (Gold et al., 1988).
- Fear of losing opioid substitution therapy - “What will I do without methadone?”; “What will I replace it with?” and “missing the dependency on or the ‘crutch’ of methadone.” (Gold et al., 1988).
- Fear of physical withdrawal - sleeping problems, back and joint aches, stomach problems, and muscle spasms. (Gold et al., 1988).
- Belief methadone provides life stability (Gold et al., 1988).
- Patients with detoxification phobia make fewer detoxification attempts (Milby et al., 1994).
- History of intravenous heroin use or intranasal heroin use as the primary opioid used (Mannelli et al., 2022).
- Anxieties about impending discomfort (Wermuth et al., 1987).
- Fear of relapse (Wermuth et al., 1987).
- Fear of failure of detox (Wermuth et al., 1987).
- Little confidence in their ability to remain abstinent (Wermuth et al., 1987).
- Pressure to not detox from family and friends/ pressure to stay on methadone (Wermuth et al., 1987).
- Not influenced by others to quit methadone (Hiltunen & Eklund, 2002).
- Long history of opiate use before Methadone Maintenance Treatment (Hiltunen & Eklund, 2002).
- History of mixed drug use prior to Methadone Maintenance Treatment (Hiltunen & Eklund, 2002).
- Higher methadone dose prior to beginning detox (Hiltunen & Eklund, 2002).
- Lower degree of anticipated abstinence symptoms. (Hiltunen & Eklund, 2002). Those undertaking detox had overcome / managed their worries/fear, but those that were not detoxing, had not done this ‘work’/changed their perception.
- Belief they need methadone - 'not possible to live without methadone' (Hiltunen & Eklund, 2002).
- Knew others who had failed to quit Methadone Maintenance Treatment (Hiltunen & Eklund, 2002).
- Low confidence in succeeding (Hiltunen & Eklund, 2002).
- Desire to postpone the problem (Hiltunen & Eklund, 2002).
- Fear of losing Methadone Maintenance Treatment (Hiltunen & Eklund, 2002).
- No support (Hiltunen & Eklund, 2002).
- Expectation/ fear of psychological abstinence (Hiltunen & Eklund, 2002).
- Expectation/ fear of physical abstinence (Hiltunen & Eklund, 2002).
- Working difficulties (Hiltunen & Eklund, 2002).
- Lack of motivation (Hiltunen & Eklund, 2002).
- Not wanting to get rid of methadone (Hiltunen & Eklund, 2002).
- More positive leisure situation (active leisure, no involvement with drug subculture) (Hiltunen & Eklund, 2002).
- More involved and more satisfied with their involvement in the drug subculture (Hiltunen & Eklund, 2002).
- Those satisfied with drug use situation were less inclined to make a withdrawal attempt (Hiltunen & Eklund, 2002).
- Low capacity at different types of treatment facilities (Stewart et al., 2000).
- Long waiting lists at facilities including detox facilities (Stewart et al., 2000).
- Negative reaction from counsellors that it was not possible to leave OMT and remain stably drug-free. “I think everyone was a bit afraid of it. Because every time I brought it up, it was a bit like that - yes, it will probably work but are you sure that you want this. And I understand that they ask those questions.” (Nesvag et al., 2024).
- Incarceration and "detox" at the same time was overwhelming. "All those girls are just going through emotional hell, and I can't see how that could in any way - that experience could help anyone. It'd have to cause more trouble. You know?" (Aronowitz & Laurent, 2016).
- Pressure to remain on opioid substitution therapy from family who feared they relapse (Nesvag et al., 2024).
- Lower quality of life (Hiltunen et al., 2011).
- Fear of destabilization (from life, physical health, mental health) – I hate to think about it (reduction) – “I would be in such fear and trepidation as to what really could happen; the pitfalls where I could end up and back to square one. It is frightening.” (Notley et al., 2015).
- Content on opioid substitution therapy (Notley et al., 2015).
- Low sense of personal efficacy in terms of ability to maintain abstinence (Notley et al., 2015).
- Identify as a drug user. ‘I have taken drugs all my life; it has just escalated from C to B to A as I do, and you know, I probably will always use heroin’ (Notley et al., 2015)
- Dual diagnosis of mental health – ‘My illicit drug use wasn’t very long, compared to how long I have been on this (methadone). And every time I think of coming off, or start to wean down, that anxiety kicks in.’ (Notley et al., 2015).
- Fear of experiencing opiate withdrawal – ‘The methadone has been in my system for so long now the withdrawal is going to be—and I know it is going to be really, really bad.’ (Notley et al., 2015).
- Mixing with the same crowd/ Drug using social group, particularly sexual relationships with other drug users – ‘It is everything; everywhere, everything, every call that comes in, everyone that walks through the door now, everyone I literally stop to talk to, I know takes drugs.’ (Notley et al., 2015).
- Fear of adverse impact on children - ‘For the children I have got to be right, they don’t want to see their dad having like it once was (being chaotic). And at my age as well that would upset their lives’. (Notley et al., 2015).
- Poor housing (Notley et al., 2015).
- Unemployment (Notley et al., 2015).
- Worry about symptoms and opioid relapse inversely associated with self-efficacy to quit Buprenorphine (Stein et al., 2019).
- Compared to those who did not try, those who tried to detox had negative expectations beforehand about the intensity of withdrawal which significantly exceed the later, actual experience. The negative expectations were less than for those who did not try to detox (Eklund et al., 1997).

**1B. Summarised Barriers to Initiation**

- Fear of return of negative emotions.
- Low patient desire for detoxification.
- Low patient recovery capital that is not supported.
- Lack of psychosocial support.
- Staff not advocating for detox of clients.
- Fear of not having opioid substitution therapy medication (n = 3).
- Availability of illicit drugs/lack of drug-free environment.
- Lack of psychological support.
- Detox not supported by staff despite patient desire.
- Fear of being left alone after detox.
- Fear of withdrawal discomfort.
- Fear of increased pain.
- Fear of return to heroin use.
- Fear of “life becoming a mess.”
- Fear they’ll return to crime.
- Fear of loss of contact with clinic.
- Concerns about overdose.
- Fear of returning to illicit drug use.
- Fear of psychological difficulties - fear/anticipation of feeling sick, irritable, in pain, anxiety, stress, nervousness, mood swings, resurfacing of feelings, and having one’s mind constantly occupied with tapering.
- Fear of physical withdrawal - sleeping problems, back and joint aches, stomach problems, and muscle spasms.
- Belief methadone provides life stability.
- Detoxification phobia.
- History of intravenous heroin use or intranasal heroin use as the primary opioid used.
- Anxieties about impending discomfort.
- Fear of relapse.
- Fear of failure of detox.
- Little confidence in their ability to remain abstinent.
- Pressure to not detox from family and friends/ pressure to stay on methadone.
- Not influenced by others to quit methadone.
- Long history of opiate use before Methadone Maintenance Treatment.
- History of mixed drug use prior to Methadone Maintenance Treatment.
- Higher methadone dose prior to beginning detox.
- Lower degree of anticipated abstinence symptoms.
- Belief they need methadone.
- Knew others who had failed to quit methadone maintenance treatment.
- Low confidence in succeeding.
- Desire to postpone the problem.
- Fear of losing methadone maintenance treatment.
- No support.
- Expectation/ fear of psychological abstinence.
- Expectation/ fear of physical abstinence.
- Working difficulties.
- Lack of motivation.
- Not wanting to get rid of methadone.
- More positive leisure situation.
- More involved and more satisfied with their involvement in the drug subculture.
- Those satisfied with drug use situation were less inclined to make a withdrawal attempt.
- Low capacity at different types of treatment facilities.
- Long waiting lists at facilities including detox facilities.
- Negative reaction from counsellors that it was not possible to leave OMT and remain stably drug-free.
- Incarceration and "detox" at the same time was overwhelming.
- Pressure to remain on opioid substitution therapy from family who feared they relapse.
- Lower quality of life.
- Fear of destabilization (from life, physical health, psychological).
- Content on opioid substitution therapy.
- Low sense of personal efficacy.
- Identify as a drug user.
- Dual diagnosis of mental health.
- Fear of experiencing opiate withdrawal.
- Mixing with the same crowd/drug-using social group, particularly sexual relationships with other drug users.
- Fear of adverse impact of detox process on children.
- Poor housing.
- Unemployment.
- Worry about symptoms and opioid relapse inversely associated with self-efficacy to quit Buprenorphine.
- Negative expectations beforehand about the intensity of withdrawal which significantly exceed the later, actual experience.

**2A. Facilitators to Initiation**

- Not wanting to be dependent on Buprenorphine - “I don’t want to have to be dependent on a chemical to regulate my world.” (Wyse et al., 2023).
- Desire to avoid side effects. Includes flattened emotional response “…there’s a desensitization of emotions, being on this medication…Like, it’s a lot harder for me to cry, it’s a lot harder for me to be empathetic...There’s just kind of a nonchalance to it, that I feel is kind of like a plateau.” His dulled emotional response also affected his family, “It sucks for my wife. It sucks for everyone around [me] because they can’t tell if I’m actually happy or not”. Also included dreams – “I used to have really vivid and prophetic dreams all the time. I never really had any surprises in my life because I always knew ahead of time with my dreams. Buprenorphine took that away, completely gone… I really wish I had my dreams back…” Desire to avoid side effects of Buprenorphine (most distressing reported were related to sleep, dreams and emotional blunted).
- In Type D DRW, opioid substitution therapy recipients’ willingness to detoxify appeared to be supported by 1. Staff who recognised the scale of the challenge presented by detoxification and purposefully housed ‘stabilising populations’ of fully abstinent prisoners (Page et al., 2016). Prison staff who recognised scale of challenge presented by detoxification, stable housing with other prisoners who were abstinent.
- All participants, except one person who had joined the program only 14 months earlier, had presently or at some point had a wish to end treatment - ‘I go to bed every night at 7.30 (...) You lose that fire inside you. That’s what’s negative, and that’s why I want to quit now. Because I want a functioning sex life. I want that fire and spark back. (#2). They described wanting to liberate themselves from their dependence on the medication and the controlled life within OAT. Vigorous terms like “a chemical leg iron”, being “tied up”, or “locked up in every way” were used." (Nehlin et al, 2022). Desire to be liberated from OAT/ not locked up.
- Side effects of the medication, such as tiredness, drowsiness, and sweating were brought up as reasons for considering leaving the program. Disturbances in their sex life were also reported. ‘I got more and more troubled by side effects. If it wasn’t for the side effects, I probably would have continued [with OAT]. Now that I’m off, I feel better. I don’t sweat; I don’t get formications [stinging sensations]. It’s very nice to wake up in the morning and not feel any formications. Or in the middle of the night. (#15). Desire to avoid side-effects of OAT.
- One reoccurring recommendation from patients was to investigate the patient’s attitude to treatment length, not only at the beginning of the treatment but also continuously. ‘That’s something they bring up at the first meeting, but then they never follow it up. It only comes up again if I bring it up myself. They always seem to find some reason why it’s not a good idea. I have never gotten to hear ‘This is what you can do, these are the alternatives...’ It’s only about plans I have come up with myself’ (#6). Continual discussions with patient about detox throughout treatment.
- One participant pointed out that staff could stimulate people to try to come off OAT by emphasizing and supporting patient control: ‘One mustn’t feel a pressure from program staff to quit, you should make your own decision. And that’s how it’s been: every lowering of the dose has been on my initiative. And beyond that I have also demanded a guarantee, that if you step off and it doesn’t work, that you can return immediately. Without having to be referred to a waiting list for months or more. Because if you should get that feeling that it is not going to work, then it might get very critical to get back into safety again’ (#9). Shouldn’t feel pressure from staff to detox, should be own decision.
- Participants recurrently underlined the need to feel in control of the speed of the detoxification and the need to feel sure that they would not be expelled if they failed.
- ‘If you made the patients feel more in control of the treatment, if you could make them less exposed to arbitrariness and predomination and such, then people may dare to try [to stop]. Because if you feel insecure, you cling to what you’ve got. Because you know you can lose it. But if it’s not that way, who knows what can happen? (#5)’ Desire for control over speed their own detox.
- High interest in undergoing detoxification was associated with having discussed coming off treatment with a greater number of categories of people in the preceding 6 months (OR = 1.72, p = 0.003), not citing concern about heroin relapse (OR = 3.18, p = 0.01), and shorter duration of current treatment episode. (Winstock et al., 2011).
- Desire to be drug free (Gold et al., 1988).
- Dislike of coming to the clinic regularly (Gold et al., 1988).
- Desire for personal growth (Gold et al., 1988).
- Having outgrown the need for methadone (Gold et al., 1988).
- Dislike of the physical side effects of methadone (Gold et al., 1988).
- Seeking to be opioid-free (Manelli et al., 2022).
- Tired of daily pill taking (Manelli et al., 2022).
- Side effects from Buprenorphine - sweats/chills. dizziness/light-headedness, drowsiness/sleepiness, and mental slowing. (Manelli et al., 2022).
- Still experiencing craving for opioids while on Buprenorphine (Manelli et al., 2022).
- Using opioids/lapses while on Buprenorphine (Manelli et al., 2022).
- Work-related concerns being on Buprenorphine (Manelli et al., 2022).
- Childcare related concerns being on Buprenorphine (Manelli et al., 2022).
- Transportation involved with Buprenorphine is too inconvenient (Manelli et al., 2022).
- Hassle of filling prescriptions (Manelli et al., 2022).
- Concerns about Buprenorphine being lost/stolen (Manelli et al., 2022).
- Buprenorphine requires too many appointments (Manelli et al., 2022).
- Cost considerations of Buprenorphine (Manelli et al., 2022).
- Occupational status increases likelihood of WA (Cushman, 1974)
- Longing for freedom from taking medication and logistics associated with medication - 'Freedom! Free to be more than a LAR patient. To be free to travel. To visit my son in another part of Norway, even on short notice. Not to have to go to the pharmacy every day, even on Sundays. I’m completely fed up - I’m so fed up with taking medicine.' (Nesvag et al., 2024).
- Self-motivation – ‘There is no good anyone trying to do it for other people ...you can only do it for yourself, otherwise it doesn’t work.’. (Notley et al., 2015).
- Encouragement from partner also in recovery/treatment. (Notley et al., 2015).
- Ready for detoxification (Cushman et al., 1973).
- Certain that no return to heroin would occur (Cushman et al., 1973).
- Repeatedly asked for detoxification (Cushman et al., 1973).
- Economic opportunities (Cushman et al., 1973).
- Desire to travel (Cushman et al., 1973).
- Sexual problems on methadone (Cushman et al., 1973).
- Attendance at the methadone clinic a nuisance (Cushman et al., 1973).
- Initiative from the clinical which the patient acquiesced (Cushman et al., 1973).
- To end physical dependence - “There's nothing I want to take every day of my life and be dependent on, not dependent, but rely on. So, I worry about missing it... I just don’t want to be stuck on anything, even anti-depressants or anything like vitamins. I really don’t want to take every day.” (Randall‐Kosich et al., 2020).
- To end need to take medication regularly (Randall‐Kosich et al., 2020).
- inconvenient visits to healthcare practitioner (Randall‐Kosich et al., 2020).
- External pressure – family, peer support groups, healthcare professionals, criminal justice administrators. (Randall‐Kosich et al., 2020).
- Stigma around medication (Randall‐Kosich et al., 2020).
- Not wanting to tell employers - “I’m afraid that I won’t be able to get a job because of it [methadone]… If it wasn’t stigmatized, I probably would stay on it.” (Randall‐Kosich et al., 2020).
- Avoid side effects of methadone/ methadone damaging their body, including sweating, fatigue, falling asleep, and euphoria - “I was nodding an awful lot, like I couldn’t stay awake even though I was on a stable dose for years and … sweating and all that. Since I’ve come down, I’m not nodding off anymore. I’m not getting super sleepy. The sweating has decreased tremendously.” (Randall‐Kosich et al., 2020).
- Desire to see if they could maintain sobriety on their own without the use of any medication (Randall‐Kosich et al., 2020).

**2B. Summarised Facilitators to Initiation**

- Not wanting to be dependent on Buprenorphine.
- Desire to avoid side effects of opioid substitution therapy (n = 2).
- Prison staff who recognised scale of challenge presented by detoxification.
- Stable housing with other prisoners who were abstinent.
- Desire to be liberated from OAT/ not locked up.
- Continual discussions with patient about detox throughout treatment.
- Shouldn’t feel pressure from staff to detox, should be own decision.
- Desire for control over speed their own detox.
- Detox discussed with a greater number of categories of people in the preceding six months.
- Not citing concern about heroin relapse.
- Shorter duration of current treatment episode.
- Desire to be drug free.
- Dislike of coming to the clinic regularly.
- Desire for personal growth.
- Having outgrown the need for methadone.
- Dislike of the physical side effects of methadone.
- Seeking to be opioid-free.
- Tired of daily pill taking.
- Side effects from Buprenorphine
- Still experiencing craving for opioids while on Buprenorphine.
- Using opioids/lapses while on Buprenorphine.
- Work-related concerns being on Buprenorphine.
- Childcare related concerns being on Buprenorphine.
- Transportation involved with Buprenorphine is too inconvenient.
- Hassle of filling prescriptions.
- Concerns about Buprenorphine being lost/stolen.
- Buprenorphine requires too many appointments.
- Cost considerations of Buprenorphine.
- Occupational status.
- Longing from freedom from taking medication and logistics associated with medication.
- Self-motivation.
- Encouragement from partner also in recovery/treatment.
- Ready for detoxification.
- Certain that no return to heroin would occur.
- Repeatedly asked for detoxification.
- Economic opportunities.
- Desire to travel.
- Sexual problems on methadone.
- Attendance at the methadone clinic a nuisance.
- Initiative from the clinical which the patient acquiesced.
- To end physical dependence.
- To end need to take medication regularly.
- Inconvenient visits to healthcare practitioner.
- Stigma around being on opioid substitution therapy.
- Not wanting to tell employers.
- External pressure – family, peer support groups, healthcare professionals, criminal justice administrators.
- Avoid side effects of methadone.
- Desire to see if could maintain sobriety on their own without the use of any medication.

**3A. Barriers to Completion**

- Detox group requested dose increase, where sleep problems and withdrawal sickness were reported as increasing, in comparison to methadone maintenance treatment group with showed slight decrease (p = 0.03). Significant differences in craving (detox> methadone maintenance treatment, p < 0.05) and % of people satisfied with dose between groups during the final month (detox> methadone maintenance treatment, p < 0.05) (Mintz et al., 1975). Withdrawal symptoms – sleep problems, sickness. Craving. Dissatisfaction with dose.
- Participants describe past attempts to stop taking opioids without the support of medication as excruciating, "*not only do you actually feel like you’re going to die, or that you would like it to end, but you know there's only one thing on this planet that will make you feel better. And you can't have it"* (Wyse et al., 2023). Withdrawal symptoms without support of medication.
- Reasons for discharge from detoxification included continued withdrawal symptoms, attributed to Subutex, and family pressures (Shaw et al., 2010). Subutex withdrawal symptoms.
- Family pressures.
- 20/30 stopped methadone taper for following reasons: feeling unstable/ withdrawal symptoms (n = 4), on top drug use/ positive urinalysis (n = 12), psychiatric instability (n = 3), and pain management (n = 1) (Calsyn et al., 2006).
- Participants expressed their reasons for failure as follows: I was afraid; I got too nervous; I couldn’t think; I couldn’t work (Berger & Schwegler, 1973).
- Factors significantly associated with treatment failure were presence of legal problems (p = 0.026), past or current cocaine abuse (p = 0.044, p = 0.027 respectively), and past hallucinogen use (p = 0.007) (Broers et al., 2000).
- All three study non-completers were discontinued from the protocol because of their inability to tolerate the withdrawal symptoms (Levin et al., 1997).
- Probability of dropout was inversely related to methadone dosage. Difficulty at low methadone dose (Fulwiler et al., 1979).
- Personal fear of relapse to drug abuse (Gold et al., 1988).
- Physical withdrawal difficulties (Gold et al., 1988).
- Social or environmental factors (Gold et al., 1988).
- Participants felt anxiety and stress interfered with attempts to taper - *"the last time it was my intention to get off, but when I got to 50 mg, I got shaky, crazy. I didn't think I would make it anyway. At the back of my mind I was thinking of getting my dose down to about 15 or 20 mg, so that I would get high off heroin again”.* (Gold et al., 1988).
- Desire or craving for drugs (Gold et al., 1988).
- Difficulties adjusting to a low dose (Gold et al., 1988).
- The drug environment - the city, crime, drug access, drug-using friends, the lack of non-using support systems (Gold et al., 1988).
- Problem with facing responsibilities - *'The sickness, the sleeplessness, the nausea, and the general discomfort is all hard. But even more important-the fears and phobias that you originally tried to smother with heroin and later methadone are still there and you have to deal with them.”* (Gold et al., 1988).
- Doubt that motivation might not be strong enough (Gold et al., 1988).
- Doubt that they might not be ready to get off methadone (Gold et al., 1988).
- Staff mentioned physical and psychological factors (Gold et al., 1988).
- Resurfacing of feelings – anxiety, stress, nervousness, mood swings, and having one’s mind constantly occupied with tapering (Gold et al., 1988).
- Fear of loneliness because feel need to relocate to be drug-free (Gold et al., 1988) - *'I think loneliness, because if I was to do all that, stay clean for the year...I would have to meet new people. To stay clean I would have to move out of that drug environment, drugs everywhere, so I would have to start all over again, with people. So I guess loneliness scares me’*.
- Emergence of clinically significant symptoms of dysphoria (Kanof et al., 1993).
- Subjective and objective withdrawal symptoms (Kanof et al., 1993).
- Fear of withdrawal symptoms emerging (Kahn et al., 1976).
- Wanting to use on top (Kahn et al., 1976).
- Problems with intimacy (Wermuth et al., 1987).
- Problems with social isolation (Wermuth et al., 1987).
- Problems with identity (Wermuth et al., 1987).
- Problems with dependency (Wermuth et al., 1987).
- Withdrawal symptoms (Cushman, 1974).
- Insufficient methadone, "not being held" (Cushman, 1974).
- Shorter duration of time spent in detoxification (Cushman, 1974).
- Physical withdrawal symptoms - *"every single day was just dreadful...like I'd get so hot that I'd sweat...I'd shiver and when I was throwing up I wasn't just throwing up anything. Just foam. Just big things of foam and it was nasty."* (Aronowitz & Laurent, 2016).
- Detox is emotionally taxing - *"I felt like it screwed my head up afterwards for a while. I didn't feel clear for a long time...I felt like I was nursing my mind and my body back to health slowly.... It’s a horrible experience. It's really - emotionally it's even harder. I can take the sickness , but you know you're in jail and you've lost everything again and you're - I feel like I'm always like - my body is an open wound emotionally...Then I just starting thinking about my kids and it's just...it's a dreary place to be"* (Aronowitz & Laurent, 2016).
- Lack of support or understanding from medical staff in prison. “*The nurses knew nothing about when I was being tapered down, they didn't come and check. Nobody came to check nothing". "They just sat there and said, 'suck it up, it's not that bad'. "The medical staff is horrible...their opinion is, you can't die from opiate withdrawal so suffer"* (Aronowitz & Laurent, 2016).
- Lack of psychological support in prison*. "I told someone...a person that you were allowed to talk to if you were on drugs and you'd get to see them once a week for half an hour and you'd tell them how you're doing and stuff like that…she was just like 'that doesn't happen', just kind of like brushed it off her should like it was no big deal"* (Aronowitz & Laurent, 2016).
- Availability of illicit substances/opioids in prison. *"that's what most people know now...they know they're not going to get their medicine so [they smuggle it in]. You'd be crazy not to take care of yourself...If I was ever in trouble again, I would bring [drugs] in because I would never go through that again. It was awful."* (Aronowitz & Laurent, 2016).
- Lack of comprehensive medical care (Aronowitz & Laurent, 2016).
- Patients felt professionals were not competent in detox from maintenance medications (Nesvag et al., 2024).
- Patients wanted extra support at last part of tapering (Nesvag et al., 2024).
- Fear of relapse to illegal drug use *- 'I knew it was going to be difficult but not that the addict in me would grow that much again. That the troll would come up like that again as soon as I got withdrawal symptoms. It still came as a shock, even though I knew it rationally, right? If someone had come here and put heroin or something on the table, I would have taken it straight away'* (Nesvag et al., 2024).
- Detoxification unit shared by illicit drug users tapering/ Detoxification unit not suitable for opioid substitution therapy detox needs. (Nesvag et al., 2024).
- Withdrawal symptoms, especially at low doses. - sleeping problems, and both somatic health issues and mental health issues (Nesvag et al., 2024).
- Withdrawal symptoms (Gossop et al., 1986).
- Withdrawal symptom intensity at low doses (Gossop et al., 1987).
- Pressures related to life situation (Gossop et al., 1986).
- The influence of other drug users (Gossop et al., 1986).
- Anxiety over methadone dose/ feeling like they couldn’t make it without methadone (Cushman et al., 1973).
- Withdrawal syndrome - irritation, sleep disturbance, restlessness, pain, fatigue, malaise, narcotic hunger, lacrimation, and GI hyperactivity (Cushman et al., 1973).
- Resurgence of difficult emotions/ traumatic memories – *‘So I wasn’t quite in reality anyway, and I could feel myself slowly coming down to reality, as I was dropping down even through the 5 mils...I could feel myself coming back with the touch, and with my feelings and everything coming back, you see the numbness out of your body going...but then with that you have got all the emotional upset coming back. And after walking around, with really a blanket over you for years, and not having to take any emotional bombardment on your life, on your brain, it is quite hard to deal with.’* (Notley et al., 2015).

**3B. Summarised Barriers to Completion**

- Withdrawal symptoms – sleep problems, sickness.
- Craving for opioids.
- Dissatisfaction with dose.
- Withdrawal symptoms without support of medication.
- Subutex withdrawal symptoms.
- Family pressures.
- Feeling unstable and withdrawal symptoms.
- Psychiatric instability.
- Pain management.
- “I was afraid.”
- “I got too nervous.”
- “I couldn’t think”
- “I couldn’t work.”
- Presence of legal problems.
- Past or current cocaine abuse.
- Past hallucinogen use.
- Inability to tolerate the withdrawal symptoms.
- Difficulty at low doses.
- Personal fear of relapse to drug abuse.
- Physical withdrawal difficulties.
- Social or environmental causes.
- Patients felt and stress interfered with attempts to taper.
- Desire or craving for drugs.
- Difficulties adjusting to a low dose.
- The drug environment - the city, crime, drug access, drug-using friends, the lack of non-using support systems.
- Problem with facing responsibilities.
- Doubt that motivation might not be strong enough.
- Doubt that they might not be ready to get off methadone.
- Staff mentioned physical and psychological factors.
- Resurfacing of feelings – anxiety, stress, nervousness, mood swings, and having one’s mind constantly occupied with tapering.
- Fear of loneliness because feel need to relocate to be drug-free.
- Emergence of clinically significant symptoms of dysphoria.
- Subjective and objective withdrawal symptoms.
- Fear of withdrawal symptoms emerging.
- Wanting to use on top.
- Problems with intimacy.
- Problems with social isolation.
- Problems with identity.
- Problems with dependency.
- Withdrawal symptoms.
- Insufficient methadone/not being held
- Shorter duration of time spent in detoxification.
- Physical withdrawal symptoms.
- Detox is emotionally taxing.
- Lack of support/ understanding from medical staff in prison.
- Lack of psychological support in prison.
- Availability of illicit substances/opioids in prison.
- Lack of comprehensive medical care.
- Patients felt professionals were not competent in detox from maintenance medications
- Patients wanted extra support at last part of tapering.
- Detoxification unit shared by illicit drug users tapering. The detoxification unit not suitable for opioid substitution therapy detox needs.
- Withdrawal symptoms, especially at low doses. - sleeping problems, and both somatic health issues and mental health issues.
- Withdrawal symptoms.
- Withdrawal symptom intensity at low doses.
- Pressures related to life situation.
- Influence of other drug users.
- Anxiety over methadone dose/feeling like they couldn’t make it without methadone
- Withdrawal syndrome - irritation, sleep disturbance, restlessness, pain, fatigue, malaise, narcotic hunger, lacrimation, and gastrointestinal hyperactivity).
- Resurgence of difficult emotions and traumatic memories.

**4A. Facilitators to Completion**

- Patients who stayed in the study reported significantly less stress and fewer sleep problems during the baseline period (detox and maintenance group). (Mintz et al, 1975). Less stress before detox, fewer sleep problems before detox.
- Completers of detox were older (36 vs 31 years mean, p < 0.01) and were more likely to have a structured inpatient/ residential treatment post-detoxification plan rather than an outpatient detoxification plan (p = 0.08) (Hakannson et al., 2014). Older age. Inpatient post-detoxification plan.
- Participants considered the one-to-one support (*‘I have never had a detox experience like I had in here. I have walked out of other detoxifications time and time again due to other people. The one-to-one worked’*), inpatient model (*‘I felt more protected here than when doing a home detox. There were no outside influences’*), patient personal responsibility for detoxification *(‘I expect to be encouraged to be responsible for my own recovery & medication’) (‘The individual basis puts you in a position where the only person you can let down is yourself. You can’t give yourself that alibi unless you’ve got someone to provide it’.),* and the open and supportive staff (*‘It was good being able to speak freely without being judged’*) as significant contributions to their detox success (Shaw et al., 2010).
- Type D (the power of the few, 5% prison population) Drug Recovery Wings. Working with very small cohorts, staff in Type D DRWs felt that they were particularly successful at supporting former heroin users through opioid substitution therapy detoxification (completion rates were as high as 90%) (Page et al., 2016). Small cohorts so staff can successfully support.
- Professional support was both intensive (a minimum of nineteen hours per week, mostly comprising group treatment), and geared towards the development of strong peer groups: all Type D DRW prisoners undertook treatment together Intensive professional support. Group treatment geared towards development of strong peer groups.
- Sharing both group therapy and accommodation seemed to support the emergence of safe therapeutic spaces. Emotional vulnerability, a sign of weakness on other wings, became a source of shared strength and personal development. *‘[Victim awareness] was stressful, it was upsetting, I did cry. [But] we’ve got nine other guys who… were looking at it cold stone sober. We were there to support each other. If it was just one on one, I think I would have gone away and got really depressed… but doing it within the group… really helped (Prisoner, Prison 8)’.* Supportive recovery environment in prison.
- Prisoners presented their peers as the most essential element of their recovery communities. Detoxification was supported in ways that seemed unimaginable on other wings: *Upstairs… people come in, “oh, why don’t you go and get this… they’ll sort you out [with drugs].” Down here, they were coming in and talking to me [when] I was lying on the bed. “You know, you’ll be alright” (Prisoner, Prison 7).* Peer support.
- The factors that worked for successful voluntary detoxification were: Generally steady employment; No welfare history; Middle class background; Majority are high school graduates; Majority have arrest records, but only 4 have convictions; Generally clean urine reports; Cooperative with program regulations; Apparently good relationships with counsellors; Younger short-term addict (Berger & Schwegler, 1973).
- Opiate users who had never injected were twice as likely to have a successful detoxification outcome (AOR 2.25 (1.20–4.25), p = 0.01) (Mullen et al., 2020). Not having history of injecting opioids.
- Receiving some inpatient treatment as part of the detoxification programme resulted in 56.3% completing a detoxification compared to outpatient only treatment (21%). So, users having any inpatient treatment included in their detoxification programme was 6 times as likely to have a successful outcome (AOR = 5.9 (2.63–13.64), p = < 0.0001) (Mullen et al., 2020). Receiving some inpatient treatment as part of a detox programme, not just outpatient only.
- Behavioural treatment, comprising a voucher incentive programme, engaging in therapeutic activities, and a community reinforcement approach (Bickel et al., 1997)
- Participants believed motivation was an important factor in tapering success (Gold et al., 1988).
- Desire to be drug-free were important factors in tapering success (Gold et al., 1988).
- Role models of others who had detoxed successfully (Gold et al., 1988) - *"They are constant source of inspiration and confidence for me. You hear so many stories of how impossible it is to detox. It's nice to see the real thing in front of you. They were successful because they desired to be free of methadone, and it was time to get complete control of their lives."*
- Staff also felt that motivation and readiness were major reasons for detox success.
- Staff felt changes in lifestyle, a good support system, self-insight, and treatment services were influential. *"The ones that I've seen do it 'successfully', the ones I haven't seen come back, have received outside support in the form of successful work situations and successful family situations like their spouse, parents, friends, and they got out of their environments. You have to get away from those people...cause they'll throw a party and wham...that's why it's very important for people to get away from the old gang.”* (Gold et al., 1988).
- Psychotherapy and counselling (both individual and group) - "*Periodic counseling would be beneficial for me. She (counselor) makes me feel like she understands me. She really is concerned. For me, that’s a definite plus."* [From both staff and clients]. (Gold et al., 1988).
- Prescription of support medication (e.g., Valium). [from both staff and clients]. (Gold et al., 1988).
- New age treatments - exercise, diet, massage, acupuncture, herbal teas. [mainly staff] (Gold et al., 1988).
- ‘Straight’ role models - 'straight' family and friends (Gold et al., 1988).
- New social associations (Gold et al., 1988).
- Taking it slow (referring to tapering process) [More staff] (Gold et al., 1988).
- Psychological coping strategies - *"keeping yourself busy", "rewarding yourself", "something to occupy one's time and mind". "Hope. The same way you handle a flu or a toothache. An addict falls into an instant gratification pattern. You realize that detoxing is not going to be painless. Reminding yourself how much you’ll hate yourself for going back can be a big help. If you keep yourself busy, you’ll stay out of trouble. I'll take any drugs that will help more than they hurt to deal with the physical discomfort."* (Gold et al., 1988).
- Hobbies for self-esteem (Gold et al., 1988).
- Support, including peer - [more staff] *"We find that a lot of them have trouble the first three or four months and that is when they need the support. They’ve been used to having the support for all the time they were on methadone, and that could be years-so to go and cut off the umbilical cord-a lot of them can’t handle it. So that should be the very first thing I would design is the aftercare to help someone who has tapered off successfully."* (Gold et al., 1988).
- Relocating one's residence - emphasised by mainly clients. (Gold et al., 1988).
- Changes to social and environmental references - Self-help groups, Narcotics Anonymous/Alcoholics Anonymous, family support, employment, relocation to living in a therapeutic community. [From both staff and clients]. (Gold et al., 1988).
- Longer length of Buprenorphine treatment prior to transition to Extended-Release Naltrexone (Manelli et al., 2022).
- Prior courses of Buprenorphine treatment were associated with higher likelihood of successful transition to Extended-Release Naltrexone (Manelli et al., 2022).
- Predictors of success were one year or longer since last use of heroin (Dawes et al., 1991).
- Little or no heroin and other hard drug use while on methadone maintenance (Dawes et al., 1991).
- Willingness to participate in a treatment recovery program (Wermuth et al., 1987).
- Non-negotiable treatment schedule is a facilitator to completion of detox (Dawes et al., 1991).
- Stronger motivation increases detox success (Cushman, 1974).
- Assimilation into nondrug world increases detox success (Cushman, 1974).
- Slower rate of detoxification (Cushman, 1974).
- Effective withdrawal treatment - *'If I hadn’t been given some special medicine to take away the tingling, I almost don’t know if I would have lasted that long. And yes, I also got benzodiazepines.'* (Nesvag et al., 2024).
- Help from friends. *I did get some information through the (municipality social service), but I got most of it through friends. They are also drug-free. Information about work possibilities and about how to get back the driver license - all the information I’ve needed to get everything; I’ve been given from them.* (Nesvag et al., 2024).
- Mental health support. *'It was when I was in (mental health) treatment that I started to get the pieces together and having a normal life.'* (Nesvag et al., 2024).
- Development of effective coping skills (Nesvag et al., 2024).
- Appropriate medical treatment (Nesvag et al., 2024).
- Social support from others (Nesvag et al., 2024).
- Long-standing contact with GP – *"I have a good relationship to my GP. He has followed me through all these years. He knows me, and I have always been honest with him, open with him about everything. So, he knows my whole life situation and my problems."* (Nesvag et al., 2024).
- Ability to temporarily stop or increase taper dose (Nesvag et al., 2024).
- Acupuncture group had greater reductions in methadone dose (Lu et al., 2024).
- Psychological support - by professionals and ex-service users – *‘There are always going to be those pinch points where, you know that stressful point where there is a crisis point...there needs to be something available for these guys, and someone they trust, and we talked about some peer support, you know peer groups run by service users or ex-service users—there is just not enough of that.’* [Commissioner]. (Notley et al., 2015).
- Having own children (Eklund et al., 1995).
- Motivation (Eklund et al., 1995).
- Staff unaware on withdrawal attempt (Eklund et al., 1995).
- Have numerous reasons to detox (Eklund et al., 1995).
- Less active leisure (Eklund et al., 1995).
- Taking medication (Eklund et al., 1995).
- Abrupt withdrawal (Eklund et al., 1995).
- Taking additional life measures (e.g. having someone look after children, time off work) to facilitate the detox process. (Eklund et al., 1995).
- Gradual detoxification had higher success rates (Senay et al., 1977).
- Self-regulated detoxification (Raynes et al., 1973).
- Subjects who expressed a clear preference for setting for detox (inpatient or outpatient) did better (58% vs 35%). (Gossop et al., 1986).
- Patients in inpatient group were more likely to complete detox compared to those in outpatient group (Gossop et al., 1986).
- Older age (Stimmel et al., 1978).
- Abused drugs for longer prior to detox (Stimmel et al., 1978).
- Male (Stimmel et al., 1978).
- Longer duration on methadone prior to detox (Stimmel et al., 1978).
- Living with family (Stimmel et al., 1978).
- Younger age (Nosyk et al., 2012).
- Male (Nosyk et al., 2012).
- Lower medical comorbidities (Nosyk et al., 2012).
- Better treatment adherence (Nosyk et al., 2012).
- Starting at lower doses of methadone (< 60 mg per day) (Nosyk et al., 2012).
- Longer tapers had substantially higher odds of success regardless of when in treatment taper was initiated (Nosyk et al., 2012).
- More gradual, stepped tapering schedule with dose decreases scheduled in only 25-50% of the weeks of taper, had greatest odds of success (Nosyk et al., 2012) .
- Detox treatment run by psychiatrist or psychologist (Chappel et al., 1973).
- Residential (inpatient) setting for detox (Chappel et al., 1973).

**5B. Summarised Facilitators to Completion**

- Less stress before detox.
- Fewer sleep problems before detox.
- Older age.
- Inpatient post-detoxification plan.
- One-to-one support model.
- Inpatient model.
- Patient personal responsibility for detoxification.
- Open and supportive staff.
- Small cohorts in prison so staff can successfully support.
- Intensive professional support.
- Group treatment geared towards development of strong peer groups.
- Supportive recovery environment in prison.
- Peer support.
- Generally steady employment.
- No welfare history.
- Middle class background.
- Majority are high school graduates.
- Majority had arrest records, but only four had convictions.
- Generally clean urine reports.
- Cooperative with program regulations.
- Apparently good relationships with counsellors.
- Younger short-term addict.
- Not having history of injecting opioids.
- Receiving some inpatient treatment as part of a detox programme, not just outpatient only.
- Behavioural treatment, comprising a voucher incentive programme, engaging in therapeutic activities, and a community reinforcement approach.
- Motivation (staff and patients highlighted).
- Desire to be drug-free.
- Role models of others who had detoxed successfully.
- Readiness.
- Changes in lifestyle.
- A good support system.
- Self-insight.
- Psychotherapy and counselling (both individual and group).
- Prescription of support medication (e.g., Valium).
- New age treatments - exercise, diet, massage, acupuncture, herbal teas.
- Support, including peer support.
- Role models - 'straight' family and friends.
- New social associations.
- Taking it slow (referring to tapering process).
- Psychological coping strategies.
- Hobbies for self-esteem.
- Support.
- Relocating one's residence - emphasised by mainly clients.
- Changes to social and environmental references - Self-help groups, Narcotics Anonymous/Alcoholics Anonymous, family support, employment, relocation to living in a therapeutic community.
- Longer length of Buprenorphine treatment prior to transition to Extended-Release Naltrexone.
- Prior courses of Buprenorphine treatment were associated with higher likelihood of successful transition to Extended-Release Naltrexone.
- Predictors of success were one year or longer since last use of heroin.
- Little or no heroin and other hard drug use while on methadone maintenance.
- Willingness to participate in a treatment recovery program.
- Non-negotiable treatment schedule is a facilitator to completion of detox.
- Stronger motivation increases detox success.
- Assimilation into nondrug world increases detox success.
- Slower rate of detoxification.
- Effective withdrawal treatment.
- Help from friends and social support from others.
- Mental health support.
- Development of effective coping skills.
- Appropriate medical treatment.
- Long-standing contact with a general practitioner.
- Ability to temporarily stop or increase taper dose.
- Acupuncture.
- Psychological support by professionals and ex-service users.
- Having own children.
- Motivation.
- Staff unaware on withdrawal attempt.
- Have numerous reasons to detox.
- Less active leisure.
- Taking medication.
- Abrupt withdrawal.
- Taking additional life measures (e.g. having someone look after children, time off work) to facilitate the detox process.
- Gradual detoxification had higher success rates.
- Self-regulated detoxification.
- Clear preference for setting for detox (inpatient or outpatient).
- Inpatient setting.
- Older age.
- Abused drugs for longer prior to detox.
- Male.
- Longer duration on methadone prior to detox.
- Living with family.
- Younger age.
- Male.
- Lower medical comorbidities.
- Better treatment adherence.
- Starting at lower doses of methadone (< 60 mg per day).
- Longer tapers.
- More gradual tapering schedule.
- Detox treatment run by psychiatrist or psychologist.
- Residential (inpatient) setting for detox.
