# Supplementary material for: Barriers and facilitators to detoxification from opioid substitution treatment: A mixed‐methods systematic review
Source: Addiction. 2026 Jun 17;121(10):2607–19. doi: 10.1111/add.70482 (PMC13578861; doi:10.1111/add.70482)
Supplement: Supplementary file 5 [file ADD-121-2607-s005.docx]

**APA PsychInfo Search Strategy**

1

exp "opioid use disorder"/

35761

2

(opi* adj4 (disorder or dependen* or addict* or abuse)).mp. [mp=title, book title, abstract, original title, name of substance word, subject heading word, floating sub-heading word, keyword heading word, organism supplementary concept word, protocol supplementary concept word, rare disease supplementary concept word, unique identifier, synonyms, population supplementary concept word, anatomy supplementary concept word]

24078

3

1 or 2

46495

4

((opi* substitution or OST or opi* maintenance or OMT or opi* agonist or OAT or opi* replacement or ORT or "medication assisted treatment" or MAT) adj4 (taper* or detox* or free or decrease* or discontin* or abstinence or cessation or withdrawal)).mp. [mp=title, book title, abstract, original title, name of substance word, subject heading word, floating sub-heading word, keyword heading word, organism supplementary concept word, protocol supplementary concept word, rare disease supplementary concept word, unique identifier, synonyms, population supplementary concept word, anatomy supplementary concept word]

934

5

(exp methadone/ or methadone.mp. or MMT.mp.) adj4 (taper* or detox* or free or decrease* or discontin* or abstinence or cessation or withdrawal).mp. [mp=title, book title, abstract, original title, name of substance word, subject heading word, floating sub-heading word, keyword heading word, organism supplementary concept word, protocol supplementary concept word, rare disease supplementary concept word, unique identifier, synonyms, population supplementary concept word, anatomy supplementary concept word]

2350

6

(exp buprenorphine/ or buprenorphine.mp. or BMT.mp.) adj4 (taper* or detox* or free or decrease* or discontin* or abstinence or cessation or withdrawal).mp. [mp=title, book title, abstract, original title, name of substance word, subject heading word, floating sub-heading word, keyword heading word, organism supplementary concept word, protocol supplementary concept word, rare disease supplementary concept word, unique identifier, synonyms, population supplementary concept word, anatomy supplementary concept word]

1603

7

4 or 5 or 6

4555

8

exp Attitudes/ or exp Cultural Attitudes/ or exp Adult Attitudes/ or exp Health Attitudes/

664407

9

(attitude* or view* or opinion* or perspective* or perception* or belief* or barrier* or imped* or facilitat* or aid* or enable* or success* or improv* or outcome* or effective).mp. [mp=title, book title, abstract, original title, name of substance word, subject heading word, floating sub-heading word, keyword heading word, organism supplementary concept word, protocol supplementary concept word, rare disease supplementary concept word, unique identifier, synonyms, population supplementary concept word, anatomy supplementary concept word]

10818780

10

8 or 9

10926090

11

3 and 7 and 10

1525

12

(mice or mouse or murine or rat* or neonatal or anaesthe* or anesthe*).mp. [mp=title, book title, abstract, original title, name of substance word, subject heading word, floating sub-heading word, keyword heading word, organism supplementary concept word, protocol supplementary concept word, rare disease supplementary concept word, unique identifier, synonyms, population supplementary concept word, anatomy supplementary concept word]

9874634

13

11 not 12

768

**MedLine Search Strategy**

1

exp Opioid-Related Disorders/

35761

2

(opi* adj4 (disorder or dependen* or addict* or abuse)).mp. [mp=title, book title, abstract, original title, name of substance word, subject heading word, floating sub-heading word, keyword heading word, organism supplementary concept word, protocol supplementary concept word, rare disease supplementary concept word, unique identifier, synonyms, population supplementary concept word, anatomy supplementary concept word]

24078

3

1 or 2

46495

4

exp Opiate Substitution Treatment/

5003

5

(opi* substitution or OST or opi* maintenance or OMT or opi* agonist or OAT or opi* replacement or ORT or "medication assisted treatment" or MAT).mp. [mp=title, book title, abstract, original title, name of substance word, subject heading word, floating sub-heading word, keyword heading word, organism supplementary concept word, protocol supplementary concept word, rare disease supplementary concept word, unique identifier, synonyms, population supplementary concept word, anatomy supplementary concept word]

39005

6

4 or 5

39005

7

exp Drug Tapering/

481

8

(taper* or detox* or free or decrease* or discontin* or abstinence or cessation or withdrawal).mp. [mp=title, book title, abstract, original title, name of substance word, subject heading word, floating sub-heading word, keyword heading word, organism supplementary concept word, protocol supplementary concept word, rare disease supplementary concept word, unique identifier, synonyms, population supplementary concept word, anatomy supplementary concept word]

4532696

9

7 or 8

4532696

10

(exp Opiate Substitution Treatment/ or (opi* substitution or OST or opi* maintenance or OMT or opi* agonist or OAT or opi* replacement or ORT or "medication assisted treatment" or MAT).mp.) adj4 (exp Drug Tapering/ or (taper* or detox* or free or decrease* or discontin* or abstinence or cessation or withdrawal).mp.)

1273

11

exp methadone/ or methadone.mp. or MMT.mp. [mp=title, book title, abstract, original title, name of substance word, subject heading word, floating sub-heading word, keyword heading word, organism supplementary concept word, protocol supplementary concept word, rare disease supplementary concept word, unique identifier, synonyms, population supplementary concept word, anatomy supplementary concept word]

22554

12

(exp methadone/ or methadone.mp. or MMT.mp.) adj4 (exp Drug Tapering/ or (taper* or detox* or free or decrease* or discontin* or abstinence or cessation or withdrawal).mp.)

2350

13

exp Buprenorphine, Naloxone Drug Combination/ or exp Buprenorphine/

7813

14

(buprenorphine or BMT).mp. [mp=title, book title, abstract, original title, name of substance word, subject heading word, floating sub-heading word, keyword heading word, organism supplementary concept word, protocol supplementary concept word, rare disease supplementary concept word, unique identifier, synonyms, population supplementary concept word, anatomy supplementary concept word]

22369

15

13 or 14

22369

16

(exp Buprenorphine, Naloxone Drug Combination/ or exp Buprenorphine/ or (buprenorphine or BMT).mp.) adj4 (exp Drug Tapering/ or (taper* or detox* or free or decrease* or discontin* or abstinence or cessation or withdrawal).mp.)

1603

17

10 or 12 or 16

4779

18

exp Attitude/

646731

19

(attitude* or view* or opinion* or perspective* or perception* or belief* or barrier* or imped* or facilitat* or aid* or enable* or success* or improv* or outcome* or effective).mp. [mp=title, book title, abstract, original title, name of substance word, subject heading word, floating sub-heading word, keyword heading word, organism supplementary concept word, protocol supplementary concept word, rare disease supplementary concept word, unique identifier, synonyms, population supplementary concept word, anatomy supplementary concept word]

10818780

20

18 or 19

10915394

21

3 and 17 and 20

1594

22

(mice or mouse or murine or rat* or neonatal or anaesthe* or anesthe*).mp. [mp=title, book title, abstract, original title, name of substance word, subject heading word, floating sub-heading word, keyword heading word, organism supplementary concept word, protocol supplementary concept word, rare disease supplementary concept word, unique identifier, synonyms, population supplementary concept word, anatomy supplementary concept word]

9874634

23

21 not 22

801

**CINAHL Search Strategy**

S1

(MM "Attitude")

S2

TI ( ((opi*) N3 (disorder or dependence or addict* or abuse)) ) OR AB ( ((opi*) N3 (disorder or dependence or addict* or abuse)) )

10,293

S3

TI ( (opi* substitution or OST or opi* maintenance or OMT or opi* agonist or OAT or opi* replacement or ORT or medication assisted treatment or MAT) N3 (taper* or detox* or free or decrease* or discontin* or abstinence or cessation or withdrawal) ) OR AB ( (opi* substitution or OST or opi* maintenance or OMT or opi* agonist or OAT or opi* replacement or ORT or medication assisted treatment or MAT) N3 (taper* or detox* or free or decrease* or discontin* or abstinence or cessation or withdrawal) )

288

S4

TI ( ((methadone or MMT) N3 (detox* or taper* or abstinence or discontin* or cessation or free or withdrawal or decrease*)) ) OR AB ( ((methadone or MMT) N3 (detox* or taper* or abstinence or discontin* or cessation or free or withdrawal or decrease*)) )

485

S5

TI ( ((buprenorphine or BMT) N3 (detox* or taper* or abstinence or discontin* or cessation or free or withdrawal or decrease*)) ) OR AB ( ((buprenorphine or BMT) N3 (detox* or taper* or abstinence or discontin* or cessation or free or withdrawal or decrease*)) )

446

S6

TI ( S1 or (attitude* or view* or perspective* or perception* or belief* or barrier* or imped* or facilitat* or aid* or enable* or success* or improv* or favourable or outcome or effectiv*) ) OR AB ( s1 or (attitude* or view* or perspective* or perception* or belief* or barrier* or imped* or facilitat* or aid* or enable* or success* or improv* or favourable or outcome or effectiv*) )

2,428,965

S7

S3 OR S4 OR S5

1,126

S8

S2 AND S6 AND S7

324

S9

S8 NOT TI ( (neonatal or rat* or mice or mouse or murine or anaesthe* or anesthe*) ) NOT AB ( (neonatal or rat* or mice or mouse or murine or anaesthe* or anesthe*) )

158

**Embase Search Strategy**

1

(opi* adj4 (disorder or dependen* or addict* or abuse)).mp. [mp=title, abstract, heading word, drug trade name, original title, device manufacturer, drug manufacturer, device trade name, keyword heading word, floating subheading word, candidate term word]

48260

2

exp opiate substitution treatment/

3379

3

(opi* substitution or OST or opi* maintenance or OMT or opi* agonist or OAT or opi* replacement or ORT or "medication assisted treatment" or MAT).mp. [mp=title, abstract, heading word, drug trade name, original title, device manufacturer, drug manufacturer, device trade name, keyword heading word, floating subheading word, candidate term word]

56049

4

2 or 3

56049

5

exp drug dose reduction/

99713

6

detoxification/ or exp drug detoxification/

37645

7

(taper* or detox* or free or decrease* or discontin* or abstinence or cessation or withdrawal).mp. [mp=title, abstract, heading word, drug trade name, original title, device manufacturer, drug manufacturer, device trade name, keyword heading word, floating subheading word, candidate term word]

6456022

8

5 or 6 or 7

6500345

9

(exp opiate substitution treatment/ or (opi* substitution or OST or opi* maintenance or OMT or opi* agonist or OAT or opi* replacement or ORT or "medication assisted treatment" or MAT).mp.) adj4 (exp drug dose reduction/ or (detoxification/ or exp drug detoxification/) or (taper* or detox* or free or decrease* or discontin* or abstinence or cessation or withdrawal).mp.)

2057

10

exp methadone/ or methadone.mp. or MMT.mp. [mp=title, abstract, heading word, drug trade name, original title, device manufacturer, drug manufacturer, device trade name, keyword heading word, floating subheading word, candidate term word]

48465

11

(exp methadone/ or methadone.mp. or MMT.mp.) adj4 (exp drug dose reduction/ or (detoxification/ or exp drug detoxification/) or (taper* or detox* or free or decrease* or discontin* or abstinence or cessation or withdrawal).mp.)

3849

12

exp buprenorphine/ or buprenorphine.mp. or BMT.mp. [mp=title, abstract, heading word, drug trade name, original title, device manufacturer, drug manufacturer, device trade name, keyword heading word, floating subheading word, candidate term word]

49009

13

(exp buprenorphine/ or buprenorphine.mp. or BMT.mp.) adj4 (exp drug dose reduction/ or (detoxification/ or exp drug detoxification/) or (taper* or detox* or free or decrease* or discontin* or abstinence or cessation or withdrawal).mp.)

2928

14

9 or 11 or 13

8087

15

exp physician attitude/ or exp attitude/ or exp patient attitude/

986151

16

(attitude* or view* or opinion* or perspective* or perception* or belief* or barrier* or imped* or facilitat* or aid* or enable* or success* or improv* or outcome* or effective).mp. [mp=title, abstract, heading word, drug trade name, original title, device manufacturer, drug manufacturer, device trade name, keyword heading word, floating subheading word, candidate term word]

14386223

17

15 or 16

14548331

18

1 and 14 and 17

2211

19

(mice or mouse or murine or rat* or neonatal or anaesthe* or anesthe*).mp. [mp=title, abstract, heading word, drug trade name, original title, device manufacturer, drug manufacturer, device trade name, keyword heading word, floating subheading word, candidate term word]

13520269

20

18 not 19

1093
